# Redefining cancer care: harnessing circulating tumor cells’ potential for improved diagnosis and prognosis

**DOI:** 10.1186/s12935-025-03883-y

**Published:** 2025-07-17

**Authors:** Divya Janjua, Apoorva Chaudhary, Udit Joshi, Tanya Tripathi, Vinita Kumar Jaggi, Alok Chandra Bharti

**Affiliations:** 1 Molecular Oncology Laboratory, Department of Zoology, University of Delhi (North Campus), Delhi, 110007 India; 2 Delhi State Cancer Institute (EAST), Government of Delhi NCR, Dilshad Garden, Delhi, 110095 India

**Keywords:** Circulating tumor cells (CTCs), Distant metastasis, Liquid biopsy, Diagnosis, Prognosis

## Abstract

Circulating tumor cells (CTCs) represent a small but clinically relevant pool of cells from tumors that can be sampled with minimally-invasive liquid biopsy procedures. They are dynamic and poorly-defined transition state of cancer cells, offering vital insights into tumor progression and metastasis. CTC frequencies are emerging as real-time means for therapeutic monitoring and patient stratification across different malignancies. However, their detection, isolation, and characterization pose a major challenge leading to variation in their counts that limit their clinical utility. This review delves into the key parameter, CTC counts, which often correlate with clinical outcomes. Further, it highlights the significance of culturing CTCs in vitro and employing CTC-derived xenograft (CDX) models to obtain in vivo insights into tumor biology, treatment efficacy, and personalized medicine strategies. The review examines the role of CTCs as diagnostic, prognostic, and therapeutic monitoring marker for different cancers. It also discusses the opportunities in CTC-directed clinical utility and associated limitations such as CTC heterogeneity and technological variations among different isolation strategies that hinder their widespread implementation. Hence, it underscores the evolving importance of CTCs in clinical oncology.

**Supplementary Information:**

The online version contains supplementary material available at 10.1186/s12935-025-03883-y.

## Introduction

Precision medicine is a realistic vision yet a milestone to achieve in medical oncology. Over the past decade, the paradigm of treatment has shifted from tissue biopsies to liquid biopsies in various solid tumors [1]. Liquid biopsies provide a minimally-invasive approach, earlier diagnosis and effective monitoring of disease progression over a prolonged period of time without physical manipulation of tumor tissues [2]. Liquid biopsy are directed primarily to detect circulating tumor cells (CTCs) [3, 4], circulating tumor DNA (ctDNA) [5], circulating cell-free DNA (ccfDNA) [6], total and cell-free circulating RNA [7], exosomal miRNAs [8], and long non coding RNAs (lncRNAs) [9]. These biomarkers reflect different aspects of the underlying disease allowing detection, identification, characterization, and monitoring of tumor progression. Despite clinical advantages different biomarkers hold, they are accompanied by their own set of limitations. ctDNA or ccfDNA do not provide information about tumor heterogeneity. High variability and RNA instability limits the reproducibility and applicability of total and cell-free circulating RNA, exosomal miRNAs or lncRNAs. In contrast, CTCs are promising tool as they allow visualization of intact cells for morphological, immunophenotypical, molecular, and functional characterization [10] and are direct representation of effected malignant tissues.

CTCs are defined as the tumor cells which disseminate from primary tumor site into the circulation, facilitated by either physical or pathological mechanisms [11]. Despite their origin from the primary tumor, these cells exhibit distinct characteristics, displaying varying degrees of stemness, undergoing epithelial-to-mesenchymal transition (EMT), and potentially harboring the ability to initiate secondary tumor growth at distant target locations [12]. The ability of CTCs to initiate secondary tumor growth is referred to as their seeding ability, based on Paget’s “seed and soil hypothesis” concerning cancer metastasis [13]. CTCs offer a live perspective into unraveling the molecular mechanisms that drive the metastatic process in solid tumors. Throughout this intricate metastatic process, CTCs exhibit diverse states, migrating either individually or in clusters [14]. This underscores the fact that CTCs constitute a rare and heterogeneous cell population within the vast sea of blood cells present in the circulation [15]. Detecting these cells in a small blood sample is comparable to locating a “needle in a haystack,” emphasizing the immense challenge posed by their rarity within the bloodstream.

CTCs serve as a potential carrier of key biomarkers offering means to explore the patient-specific mechanisms operating during metastasis and carcinogenic progression [16]. Hence, they hold considerable value as a reliable prognostic marker [17]. The CTCs numbers are showing direct association with the stages of the tumor [18] and guide therapeutic regimens [19]. These cells provide a minimally-invasive, real-time means to investigate intra- and inter-tumor heterogeneity. Despite several technological variations, they are emerging as invaluable tools to monitor disease severity and response to treatment over time [20]. The extensive investigation of CTCs permits early diagnosis of cancer [21], but the isolation and enumeration of these rare heterogeneous CTCs pose major challenge to their clinical utility.

This review is a comprehensive and integrative analysis of the clinical relevance of CTCs, with a focused evaluation on CTC enumeration and CTC culture. By systematically examining evidence from the past decade, it aims to correlate these CTC-based parameters with diagnostic, prognostic, and therapeutic outcomes in solid tumors, particularly in clinical-settings, which is currently lacking. Furthermore, it underscores both the technological advancements and persisting challenges in the field, identifying key research gaps and discussing avenues for future research directed towards translation of CTC technologies into precision oncology. The review particularly refrains from elaborating different CTC-detection platforms that can be accessed through a recent review [22]. To ensure a robust and empirical synthesis, we conducted a targeted literature search in PubMed using the following terms: Circulating tumor cells OR CTC Count OR CTC culture OR CTC-derived xenograft (CDX) models AND Diagnostic marker OR Prognostic marker OR Therapeutic monitoring marker. Hence, this review consolidates the current understanding and offers a roadmap for future research aimed at maximizing the clinical implementation of CTCs in early detection and treatment of malignancies arising of solid tumors.

## CTC counts in different malignancies

**Fig. 1:**
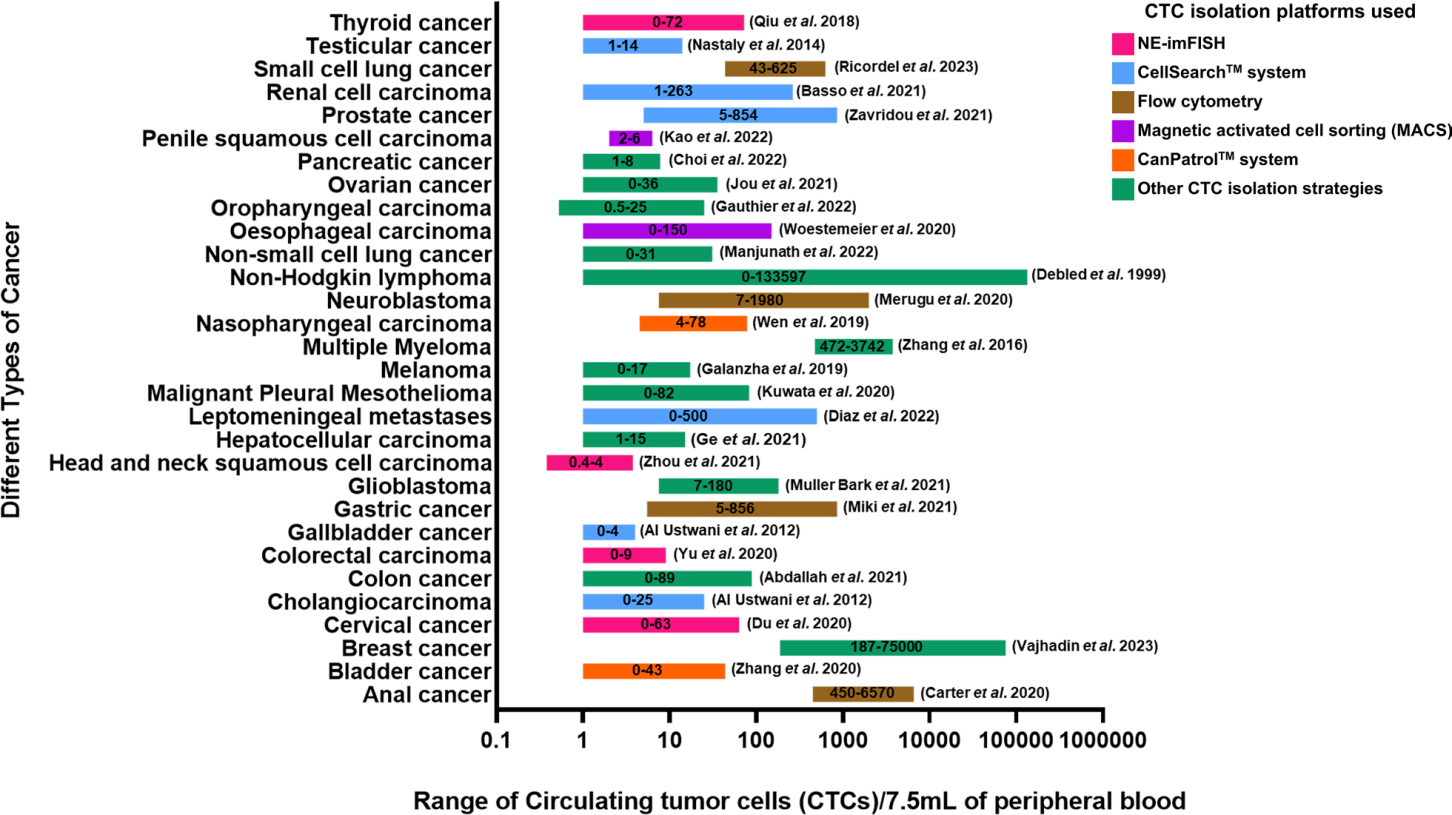
Cancer specific CTC count as reported till date. Representative plot of range of circulating tumor cells (CTCs)/ 7.5 mL of peripheral blood as isolated from different cancer types. (Disclaimer: The figure represents an illustrative synthesis rather than directly comparable values. It reflects the broad range of reported CTC counts observed over a span of different clinical stages, CTC isolation techniques, detection platforms, and statistical approaches are tabulated in Supplementary Table ST1.)

Reported CTC numbers vary greatly across different malignancies. These differences in CTC counts reflect variations related to the stage of cancer progression, specific characteristics of each malignancy, and their direct association with tumor burden. These complexities pose a great challenge for developing targeted approaches to detect and manage CTCs effectively in clinical settings. The representative studies detailed in Fig. 1 summarize a broad range of CTC numbers observed in different malignancies using different isolation platforms. Nevertheless, these findings demonstrate that presence of CTCs in circulation is a common phenomenon across diverse malignancies.

In an early study on follicular non-Hodgkin’s lymphoma, CTC counts ranged widely from 0 to 17,813 cells/mL of peripheral blood. Patients in complete remission showed marked reductions in CTCs. Detectable CTCs post-treatment correlated with relapse occurring within 4 to 11 months, serving as a predictor for relapse occurrence [23]. In gall bladder and cholangiocarcinoma, using a cutoff of 2 CTCs/7.5 mL of blood, all CTC-positive patients were of stage III or IV. Changes in serial CTC counts post-treatment correlated with clinical and radiological improvements, guiding treatment decisions and predicting outcomes [24]. Using Ficoll density gradient centrifugation, the mean CTC count was 13 in 3 × 10^6^ mononuclear cells from 9 to 17 mL of blood (range 2–60, median 8.5). The CellSearch system found a mean of 2.6 CTCs/7.5 mL of blood (range 1–14, median 1), with 20% of CTC-positive testicular cancer patients showing clusters of 3–5 cells, highlighting the impact of technological variations in isolation strategies on CTC enumeration [25].

The average counts of CD138^+^ multiple myeloma (MM) CTCs varied from 63–499 CTCs/mL. The heterogeneity among MM CTCs, both within individual patients and between different patients, was evident in the variations in cellular morphology and pS6 signal levels that allowed patient risk stratification [26]. Count of CTCs can significantly change before and after treatment, making it a valuable prognostic marker. In differentiated thyroid cancer, CTCs ≥ 5 predicted distant metastases, and ≥ 7 suggested poor response to I-131 treatment and worse prognosis in cases with distant metastasis [27]. In addition, 27 out of 28 melanoma patients showed signals indicating single CTCs, clusters, and possibly rolling CTCs [28]. Similarly, in nasopharyngeal carcinoma (NPC), CTCs were found even in early stages, with higher ratios of mesenchymal CTCs in advanced stages. CanPatrol™ CTC-enrichment technique identified 1495 CTCs, categorizing them into 190 epithelial, 1,022 hybrid, and 283 mesenchymal CTCs [29]. In anal cancer patients, 60–876 cells/mL Pan-CK^+^ CTCs were identified in 7 of 8 patients using Amnis® ImageStream®X Mk II Imaging Flowcytometry [30].

In cervical cancer (CaCx), CTCs were detected in 80% of patients. Patients with detectable CTCs had significantly shorter progression-free survival (PFS) compared to those without CTCs [31]. The E1-chip and NZ1.2-chip for microfluidic CTC detection in malignant pleural mesothelioma had median CTC counts of "0" and "2" respectively, with the NZ1.2-chip proving more effective. CTC counts were higher in advanced stages among epithelioid patients [32]. In neuroblastoma, CTCs were present in 26 out of 42 blood samples and disseminated tumor cells (DTCs) in 25 out of 35 bone marrow samples. Higher CTC counts in newly diagnosed, high-risk patients were linked to incomplete bone marrow response after initial chemotherapy [33]. In esophageal squamous cell carcinoma (ESCC), epithelial cell adhesion molecule (EpCAM)-positive and cytokeratin (CK)-positive CTCs were found in 25.6% of patients. Patients with more than 2 CTCs had shorter recurrence-free survival (RFS) and overall survival (OS) [34]. In colorectal carcinoma (CRC), the median CTC count was 2, with a positive rate of 65.8%. Recurrent patients had a higher detection rate (87.5%) compared to non-recurrent patients (59.6%). CTC levels served as a prognostic factor for RFS in stage II CRC and helped stratify patients for adjuvant chemotherapy [35]. In bladder cancer, CTCs were found in 86.3% of patients. Both non-muscle-invasive bladder cancer (NMIBC) and muscle-invasive bladder cancer (MIBC) showed distinct patterns of epithelial, mesenchymal, and stem cell marker expression. Higher levels of OCT4 and mesenchymal markers were more common in CTCs from MIBC, suggesting their role in tumor invasion and progression [36].

For colon cancer, a study of 69 stage I-III patients found a baseline median CTC level of 0–94 cells/8 mL peripheral blood with higher levels linked to circulating tumor microemboli (CTM) presence, demonstrating the utility of CTC counts in managing and stratifying colon cancer patients [37]. In a cohort of 195 metastatic renal cell carcinoma (mRCC) patients receiving sunitinib or pazopanib, CTC counts monitored using CellSearch system revealed an initial positivity of 46.7%. Patients with three or more CTCs at baseline showed significantly shorter PFS and OS. These findings underscored the potential utility of CTC counts in predicting outcomes for mRCC [38]. In hepatocellular carcinoma (HCC), CTCs were found in 27% of patients, with low counts (median of 2 cells, ranging from 1–15). Higher CTC counts were associated with poorer survival [39]. The count of EpCAM- carcinoembryonic antigen (CEA)^+^ cells was significantly higher in gastric cancer patients with stage II-III and IV compared to those with stage I. Patients with high EpCAM-CEA^+^ cell counts had a reduced 3-year RFS. Therefore, CEA^+^ CTCs emerged as a clinically valuable biomarker [40]**.** In glioblastoma, CTCs were found in 45% of patients before surgery and 58% after surgery. Patients without CTCs after surgery had notably longer RFS compared to those with > 1 CTC [41].

CTC count helps determine the range of CTCs present in the blood, providing a quantitative measure that can vary significantly among patients. In prostate cancer patients, CTCs counted using the CellSearch system, with values ranged from 5 to 854 cells/7.5 mL of blood [42]. In head and neck squamous cell carcinoma (HNSCC), 58.9% of patients had CTCs with a cutoff of 3 CTCs/mL. Higher CTC counts were observed in advanced clinical stages and were independent prognostic factors for PFS and OS [43]. In pancreatic cancer patients, higher disease stages and regional lymph node metastasis were associated with increased CTC counts in portal venous blood. Among patients with low portal venous CTC counts (≤ 2), 57.1% were in stage I. In contrast, nearly all patients with high CTC counts (≥ 3) were in stage II or III (92.9%, 13/14). Additionally, regional lymph node metastasis was significantly more prevalent in patients with high CTC counts (85.7%) compared to those with low counts (38.5%) [44]. In a study of 101 newly diagnosed leptomeningeal metastasis (LM) patients, cerebrospinal fluid CTC (CSF-CTC) counts predicted survival, with a mortality risk. CSF-CTC quantification emerge as a better prognostic tool than neuroimaging, providing a quantitative measure of CNS disease burden [45]**.** Negative enrichment of CTCs in oropharyngeal cancer using RosetteSep™ showed minimum, maximum, and median CTC counts of 0.07, 3.34, and 0.22 CTC/mL, respectively. Monitoring CTC kinetics during treatment was found to be more valuable than simply detecting CTC levels. Variations in CTC counts provided better guidance for managing treatment, potentially allowing for early adjustments in treatment strategies [46]. CTC count can significantly aid in patient stratification. In penile squamous cell carcinoma, all pre-surgery patients showed CTCs whereas at post-surgery, the counts decreased [47]. CTC count also offer critical information on tumor load and disease advancement. CD56 + CTCs were identified in small cell lung carcinoma (SCLC) using CD56 as a positive marker and CD45 as a negative marker. Higher CD56 + CTC counts at diagnosis were associated with disease spread [48]. Zinc ferrite (ZnFe_2_O_4_) magnetic nanoparticles (NPs) detected breast cancer cells with a limit of 3 cells/mL, ranging from 25 to 10^4^ cells/mL [49].

Hence, CTC levels exhibit significant variability across different malignancies, driven by tumor type, clinical stage, tumor burden, and diversity of isolation and detection techniques employed. This heterogeneity underscores the complexity of utilizing CTCs as robust biomarkers in clinical oncology. While numerous studies demonstrate strong associations between CTC counts and disease progression, treatment response, and survival outcomes, broad spectrum of available methodologies and lack of harmonization among them present challenges for their widespread clinical adoption.

## Specificity of CTC counts

CTCs offer robust diagnostic value because of their high specificity in identifying tumor-derived cells in the bloodstream. Using a microfluidic chip-based detection system, no CTCs were found in ten healthy subjects. With a cutoff of ≥ 4 CTCs in 4 mL blood, sensitivity was 62.5% and specificity was 100% in ovarian cancer [50]. Non-screened non-small cell lung carcinoma (NSCLC) patients had significantly higher CTC counts compared to patients screened through the lung cancer screening report and data system. CTCs were absent in healthy controls. CTC clusters, ranging from 0 to 1/mL of blood, were identified in non-screened NSCLC patients but were absent in high-risk and healthy controls. This highlighted the diagnostic relevance of CTC counts for NSCLC detection [51]. Similarly, in 19 healthy volunteers, no CTC-associated signals were detected within established thresholds [28].

In a study using the GILUPI CellCollector, the detection rate of CTCs was found to be 52.9% (18 out of 34) in patients diagnosed with lung cancer via pathology, compared to 10% (1 out of 10) in patients with benign disease, and no CTCs were detected in the control group [52]. Similarly, when examining the prognostic significance of CTCs in cancer with the NanoVelcro system, 72% (36 out of 50) of tongue squamous cell carcinoma patients had CTCs in their bloodstream, while no CTCs were detected in healthy individuals. CTCs proved to be an independent prognostic marker, with levels closely correlating with clinical staging, N staging, and disease progression [53]. Additionally, CTC phenotypes were present in each patient but not in the control group, showing a sensitivity of 83% and a specificity of 100% for distinguishing control from benign breast disease, and both sensitivity and specificity of 100% for distinguishing control from breast cancer without CT [54]. These findings highlight high specificity of CTC offering diagnostic accuracy in detecting residual disease across various cancers, demonstrating its critical role in personalized cancer management.

## CTC isolation and culture

In order to assess metastatic potential of CTCs, several attempts have been made to culture CTCs in vitro to study patient-specific tumor characteristics, course of cancer progression, predictive molecular pathology, and for screening drug sensitivity [55]. In one study, CTCs isolated from lung cancer patients using MetaCell technology proliferated in vitro with a success rate of 64.3% for functional characterization of CTCs [56]. Similar culture techniques have been utilized for successful culture of CTCs isolated from urinary bladder cancer [57], ovarian [58], endometrial cancer [57], NSCLC [59], as well as HCC [60, 61]. Further, culture under hypoxic and non-adherent conditions have been optimized for proliferation of CTCs ex vivo in breast cancer [62, 63] for individualized testing of drug susceptibility. The ability of CTCs to grow in culture reflects their self-seeding capacity and was inversely correlated to patient’s therapeutic response [64].

Furthermore, CTCs obtained from cancer patient were used to create CTC-derived xenografts (CDX) models in different cancers (Table 1**)**. These models offer personalized tumor representations for testing therapies and identifying mechanisms of drug resistance [65]. CDX models aim to mimic human tumor characteristics in vivo, offering insights into tumor biology, treatment efficacy, and personalized medicine approaches. These models are viable option for testing the response of treatment to some drug-resistant cancer types and allow development of targeted therapies. Nonobese diabetic (NOD)/severe combined immunodeficient (SCID)/Interleukin-2 lambda (IL2λ)-receptor null (NSG) mouse models are preferred for xenografting due to high success rate, however, other strains of mice have also been used [66]. It was observed that > 400 CTCs were sufficient for successful CDX models generation throughout. CTCs derived from the patients and CDX models were also subjected to downstream gene sequencing and transcriptomics analysis that revealed various mutations present in both the CDX model-derived CTCs and the patient-derived CTCs, allowing us to compare their expression patterns [67, 68]. It was observed that though majority of CTCs were similar in both the patient and the CDX models, but some heterogeneity between the two populations was also noted [67]. These models have demonstrated significant potential for studying cancer metastasis and tumorigenicity in vivo [69]. They could be utilized to evaluate drug responses before administration to patients as well as to study metastatic potential and aggressiveness of the tumor in the absence of the patient [70].

**Table 1:** Summary of circulating tumor cells-derived xenograft (CDX) models used as in vivo model for downstream applications

Cancer type	CTC isolation technology used	Markers used	No. of CTCs xenografted	Source of xenografted cells* (Patient details)*	Level of xenograft	Model organism	Site of Injection	Time from CTCs to Palpable Tumor *(with size)*	Success rate	REF
Small cell lung cancer (SCLC)	CellSearch™	EpCAM CK	> 400 CTCs/ 7.5 ml	n = 6 *(M:F::2:4)* *Median age* = *69y*	Primary	NOD.Cg-PrkdcscidIl2rgtm1Wjl/SzJ (NSG) mice *(8–16 week old, Female)*	Subcutaneously into flanks	2.4–4.4 months *(Doubling time: 5–21 days)* *(Volume: up to 1000 mm*^*3*^*)*	66.7%	[67]
Prostate cancer	FACS	CD45 DAPI	*Range of CTCs: 100–10,000* *(*250µL*, Regardless of CTC concentration)*	Metastatic prostate cancer patient	Primary	Immunodeficient mice *(8–10 weeks old, Male)*	Subcutaneously into both upper flanks	Euthanized when the tumor size reached a diameter of 0.5-1 cm	Successful	[66]
Breast cancer	AutoMACS system	Ep-CAM CD44 CD90	0.3–1.5 × 10^6^ CTCs/site, 20 sites/sample	n = 3	Primary	NOD-*scid Il2rgnull* mice, *(6–8 weeks of age, Female)*	Mammary fat pads	2/20 *(Frequency of tumor/site)* *Mice killed at 6 weeks/months*	33.3%	[71]
	FACS	CD34 CD105 CD90 CD73 CD45	1 × 10^6^ derived from TNBC-CDX	n = 3 *(Stage: T2N3b & IV)*	Secondary	Immunodeficient mice [NOD.Cg-Prkdcscid Il2rgtm1Wjl/SzJ (NSG)] *(4–6 week-old)*	Intracardiac injection	Euthanization done after development of any clinical symptom *(such as hunched, ruffled coat, lethargic posture, *etc*.)*	Successful	[68]
Non-small cell lung cancer (NSCLC)	CellSearch™ + RosetteSep™	EpCAM pan CK CD45 DAPI	> 400 CTCs/7.5 ml in successful CDX models	n = 1 *Stage: T1aN2M1b* *48y, Male*	Primary	CB-17/lcrHsd-*PrkdcscidLystbg-J* (SCID-*bg*) mice *(10–12 week-old, Female)*	NA	30–95 days *(Treatment started when tumor volume reached 200-250mm*^*3*^*)*	Successful *(Post-radiotherapy)*	[69]
	CellSieve™ + CellSearch™	EpCAM Pan-CK mCD45 DAPI CD45	NA	n = 10 *(M:F::7:3)* *(Range: 52-85y)*	Secondary	Naive NOD-SCID-NSG (NOD.Cg-*Prkdc*^*scid*^* Il2rg*^*tm1Wjl*^/SzJ) mice *(8–10 week-old)*	Subcutaneously into flanks	26 & 47 days respectively (Volume: up to ~ 1,000mm^3^)	20%	[70]
Pancreatic cancer	Labyrinth device	CK19 + CD45 DAPI +	10^6^ cultured CTCs	n = 3 a. *Male (53y, 14CTC/mL)* b. *Female (74y, 44CTC/mL)* c. *Female (54y, 20 CTCs/ mL)*	Primary	NOD/SCID mice *(4–5 weeks old)*	Subcutaneously into flanks	Euthanized when the tumor size reached a diameter of 1 cm	100%	[72]

*BALB* Bagg Albino mice, *CD* cluster of differentiation, *CDX* CTC-derived xenograft, *CK19* cytokeratin 19, *DAPI* 4′,6-diamidino-2-phenylindole, *EpCAM* epithelial cell adhesion molecule, *F* female, *FACS* fluorescence assisted cell sorting, *IHC* immunohistochemistry, *M* male, *MACS* magnet assisted cell sorting, *NOD* nonobese diabetic, *NSG* NOD SCID gamma mouse, *SCID* severe Combined Immunodeficient, *SCID-bg* severe combined immunodeficient beige, *T4N0M0* TNM classification of tumors of the exocrine pancreas: T4, tumor extends directly into any of the following: stomach, spleen, colon, adjacent large vessels, *N0* no regional lymph node metastasis, *M0* no distant metastasis, *TNBC* triple-negative breast cancer

CTC cultures have also been utilized for various drug screenings, to predict the susceptibility to therapeutic drugs like in SCLC patients [73]. A pressurized in vitro CTC culture platform was developed for anticancer drug screening which revealed that overexpression of ATP binding cassette subfamily C member 1 (ABCC1) reduced intracellular doxorubicin concentration, thereby, providing a novel insight into the chemoresistance mechanism of metastatic human breast cancer cells [74]. eSelect, a biomimetic cell culture system was developed, which enabled ex vivo expansion and drug sensitivity profiling of CTCs from advanced-stage HNSCC patients. Cisplatin sensitivity profiles of patient-derived CTCs expanded ex vivo correlated with clinical response to cisplatin treatment, thereby highlighting the potential of this predictive assay to guide HNSCC treatment [75]. The recent development of CTC-processing system: acoustic bubble for spheroid trapping, rotation, and culture: a tumor-on-a-chip platform (ABSTRACT) allowed successful on-chip spheroid culture which held great potential for downstream analysis of tumor cells, such as personalized drug testing and therapeutic monitoring of metastatic progression [76]. The CTC-derived organoid expansion was achieved from pancreatic ductal adenocarcinoma (PDAC) in 3 weeks, with culture efficiency of 87.8%. Moreover, the drug sensitivity profiles from these CTC-derived organoid cultures correlated meaningfully with treatment response [77]. While multiple studies have successfully cultured CTCs from diverse malignancies, variability in success rates and reliance on specialized conditions (such as hypoxia, non-adherent cultures), highlight the biological complexity of these cells. CDX models offer powerful in vivo systems to study metastatic potential, tumor evolution, and drug resistance, closely mirroring patient tumor biology. Despite demonstrating high potential for individualized treatment planning and drug screening, these systems are still in developmental stages and require further validation and standardization before they can be integrated into routine clinical practice. Moreover, these are closed and proprietary systems, which require specially-trained workforce and strong laboratory infrastructure.

## The emerging trend in CTC-directed clinical oncology

Harnessing the potential of CTCs in clinical practice has resulted in the development of CTC-directed clinical trials across different malignances as summarized in Table 2. These ongoing clinical trials have different end points ranging from studying CTC characterization and profiling, diagnosis, prognostication, therapeutic monitoring, residual cancer burden, and correlation between patient-derived organoids (PDOs) and CTCs. Among various CTC parameters, CTC count remains the most commonly analyzed, alongside other parameters like CTC characterization, CDX models, protein expression profiling, and CTC culture. Further, as CellSearch is the only Food and Drug Administration (FDA)-approved technology for isolating CTCs in breast, prostate, and colorectal cancers, most clinical trials have been disproportionately concentrated on these three cancer types. However, this technology has its own limitations as it relies on EpCAM expression, which confers it high specificity but has lower sensitivities. Many CTCs have been reported that lack or express low level of EpCAM that can go undetected as reported in breast cancer [78, 79] and gastric cancer [40]. Nevertheless, CTC-directed clinical trials are pivotal in exploring the multifaceted roles of CTCs in cancer management and provide critical insights into clinical potential of CTCs. The subsequent sections delve deeper into the already documented roles of CTCs in diagnostics, prognostics, and therapeutic monitoring. Examination of these aspects will improve the understanding on how CTCs serve as valuable biomarkers in diagnosing cancer, predicting patient outcomes, and monitoring therapeutic responses.

**Table 2:** Ongoing, active-but-not-recruiting & recruiting, clinical trials of circulating tumor cells in different cancer types

Cancer type	Phase of study	Type of clinical trial	Specificity of clinical trial	Monocentric/ Multicentric study	Main objective	Sample size (n)	CTC component used	CTC isolation/ detection strategy	Drug combination (Intervention)	Period	Target follow-up duration	End point of the study	Identifier trial no
Breast cancer	NA	Observational	Prospective Cohort study	Monocentric	Compare genetic markers on CTCs pre- and post-chemotherapy	300	CTC count CTC characterization CDX models	NA	Chemotherapy	2005–24	~ 6 years	Prognostication Therapeutic monitoring	NCT00353483
	NA	Observational	Prospective Cohort study	Monocentric	Characterization of CTCs by targeting ER, HER2, M30, VEGFR2, EGFR	280	CTC morphology & phenotype Protein expression profiling	Fiber-Optic Array Scanning Technology (FAST) CK + , CD45-, DAPI +	NA	2008–23	Post therapy: 8–12 weeks, 20–24 weeks, and at 9,12 and 24 months	CTC characterization & profiling	NCT01048918
	II	Interventional	Randomized controlled Parallel study Open-label	Multicentric	Evaluation of CTC dynamics pre- & post-treatment	116	CTC count	CTCs/7.5 mL	Ixabepilone	2009–24	At 18 weeks	RFS Prognostication Therapeutic monitoring	NCT00877500
	NA	Observational	Prospective Cohort study	Multicentric	Correlative molecular analysis of CTCs, primary and metastatic tumor samples	100	+ CTC count	NA	NA	2009–23	2 years	Prognostication	NCT00941759
	III	Interventional	Randomized controlled Double blinded Placebo Parallel study	Monocentric	Therapeutic monitoring by CTCs and their impact on survival	1100	CTC count	NA	Trastuzumab	2013–27	Up to 5 years	DFS, OS Prognostication Therapeutic monitoring	NCT01785420
	II/III	Interventional	Randomized controlled Parallel study Open-label	Multicentric	Correlation of CTCs with PFS	129	CTC count	≥ 5 CTCs/7.5 ml	Stereotactic Body Radiotherapy Surgery	2014–26	Every 3/6 months, annually	PFS Prognostication	NCT02364557
	NA	Interventional	Single arm Open-label	Monocentric	Evaluation of CTC dynamics pre- & post-therapy	23	CTC count	NA	Veliparib Lapatinib	2014–24	Cycle (28 days)	Therapeutic monitoring	NCT02158507
	II	Interventional	Prospective Open-label parallel study	Monocentric	Detect CTC prevalence, dynamics and characteristics	116	CTC count Phenotypic characterization	Hormone receptor + , HER2- CTCs	Ribociclib Eribulin	2014–24	8–12 weeks	PFS, OS, DCR CTC dynamics Prognostication	NCT02035813
	II	Interventional	Single arm Open-label	Monocentric	Evaluation of CTC dynamics pre- & post-treatment	6	CTC count	ER + : ER- CTCs	[18F]FES	2015–24	4 years	Therapeutic monitoring	NCT02409316
	III	Interventional	Randomized controlled Parallel Open-label	Monocentric	Evaluation of CTC dynamics pre- & post-treatment	270	CTC count	NA	Pertuzumab Trastuzumab Capecitabine Paclitaxel Vinorelbine Docetaxel Exemestane Letrozole Anastrozole Fulvestrant Ribociclib Nab-Paclitaxel Eribulin Leuprorelin Goserelin	2015–24	3–9 weeks	DCR, ORR, OS, PFS Prognostication Therapeutic monitoring	NCT02344472
	II	Interventional	Single arm Open-label	Monocentric	Efficiency of treatment in CTC-positive patients	100	CTC count	CellSearch	Pembrolizumab Carboplatin	2017–23	Up to 3 years	OS, PFS, ORR, CBR, TTNM Prognostication Therapeutic monitoring	NCT03213041
	II	Interventional	Single arm Open-label	Monocentric	Evaluation of PDL1 + CTC dynamics pre- & post-treatment	37	CTC characterization	PDL1 + CTCs	Atezolizumab Cobimetinib Eribulin	2017–24	Up to 2 years	ORR, DOR, CBR, OS, PFS Prognostication Therapeutic monitoring	NCT03202316
	NA	Interventional	Single arm Open-label	Multicentric	Treatment recommendation on basis of CTC dynamics	200	CTC count	NA	NA	2018–23	Up to 1 year	Prognostication Treatment regimen	NCT02965755
	NA	Observational	Retrospective Cohort study	Monocentric	Efficacy of CTCs surveillance in predicting the treatment response	1000	CTC count CTC characterization	Expression of PDL1 and FOXC2	NA	2019–29	Up to 10 years	iDFS, OS, pCR Prognostication Diagnosis Therapeutic monitoring	NCT05326295
	II	Interventional	Non-Randomized Parallel study Open-label	Multicentric	Determine proportion of patients with undetectable CTCs pre- & post-treatment	6	CTC count	NA	Atorvastatin Capecitabine	2019–23	At 2 years	RFS Prognostication Therapeutic monitoring	NCT03872388
	I (Early)	Interventional	Single arm Open-label	Monocentric	Evaluation of PDL1 + CTC dynamics pre- & post-treatment	20	CTC count	PDL1 + CTCs	Rucaparib	2019–24	6 months	Therapeutic monitoring	NCT03911453
	NA	Observational	Non-inferiority randomized controlled	Multicentric	Detection of CTCs in postoperative conditions	500	CTC count	CTCs ≥ 2 or CD133 ≥ 1	NA	2019–29	Once every 4 months in 2 years, every 6 months in 3–5 years, & yearly in > 5 years	DFS, OS Prognostication	NCT04065321
	NA	Observational	Prospective Cohort study	Multicentric	Evaluate CTC dynamics post neoadjuvant chemotherapy	200	CTC count	≤ 2 CTCs/ 2.5 mL blood or CD133 ≤ 1	Taxanes or/and anthracycline-based therapy	2019–24	NA	Therapeutic monitoring	NCT04059003
	NA	Observational	Case–control Prospective study	Monocentric	Detection of FR + CTCs as diagnostic biomarker	200	Enumeration of FR + CTCs	FR + CTCs (FU/3 ml)	NA	2020–23	3	Diagnosis	NCT05633680
	I (Early)	Interventional	Single arm therapeutic exploratory study Open-label	Monocentric	Efficacy of cardiac glycosides to disrupt CTC clusters	9	Mean CTC cluster size & no	Change in mean CTC cluster size & no. (in ng/ml)	Digoxin	2020–22	Day 0/3/7/10/14/17/21	Therapeutic monitoring Prognostication	NCT03928210
	NA	Interventional	Randomized Double-blind Parallel study	Multicentric	Detect presence of CTCs	766	CTC count	≥ 1 CTC/10 mL	Mediterranean diet supplemented with extra-virgin olive oil Low-fat diet	2020–24	1.5 years	Diagnostic Therapeutic monitoring Prognostication	NCT04174391
	III	Interventional	Randomized controlled Parallel study Open-label	Multicentric	Evaluation of CTC dynamics pre- & post-treatment and correlation with survival	378	CTC count	< 5 versus ≥ 5 CTC/7.5 mL	Paclitaxel Capecitabine Letrozole Anastrozole Fulvestrant Abemaciclib	2020–28	24 months	OS, PFS Prognostication Therapeutic monitoring	NCT04158362
	0	Observational	Prospective-explorative study Cohort study	Monocentric	Age-dependent influence of radiotherapy on CTC dynamics Correlation of CTCs Phenotype to Patient Age Comparison of phenotypic and molecular biological profile of CTCs with the corresponding characteristics of primary tumor	200	CTC count CTC genetic and phenotypic characterization	CTC count/ µL of blood	Radiation: Adjuvant radiotherapy Drug: neoadjuvant and adjuvant chemotherapy	2021–26	3 years	RFS Prognostication	NCT04902937
	II	Interventional	Single arm Open-label	Monocentric	Evaluation of PSMA expression in CTCs	15	CTC characterization	Immunofluorescence	18F-DCFPyL	2021–23	Up to 2 weeks	Diagnostic	NCT04573231
	NA	Observational	Case-only Prospective	Monocentric	Development of PDX model	100	CDX	CyTOF	NA	2021–42	Up to 12 months or max. of 20 years	Residual cancer burden Prognostication	NCT04703244
	III	Interventional	Randomized controlled Parallel Open-label	Multicentric	Utilize CTCs as biomarker for early detection of disease progression and prediction of survival	170	CTC count	NA	Radiotherapy	2021–26	2 years	PFS, OS Prognostication Diagnosis	NCT04646564
	II	Interventional	Randomized clinical trial Cohort study Factorial study design	Monocentric	Therapeutic implication of CTC count during differential HER2 positivity	80	CTC count	IHC and ISH HER2 ± CTCs	Pertuzumab Trastuzumab	2021–28	Up to 5 years	DFS, pCR Prognostication	NCT04993014
	NA	Observational	Case-only study Prospective	Multicentric	Impact of CTCs on treatment decisions, response assessment and prognosis	65	CTC count	CellSearch	NA	2022–24	18 months	PFS Prognostication Therapeutic monitoring	NCT05662345
	NA	Interventional	Prospective Non-randomized Open-label Parallel study	Multicentric	Evaluate prognostic significance of in vivo CTCs	484	CTC count	GILUPI CellCollector®	NA	2022–29	Up to 3 years	iDFS, OS, pCR Prognostication	NCT05360290
	II	Interventional	Single arm Open-label	Monocentric	Establishment of correlation of PDL1 + CTCs with prognosis	30	CTC count	PDL1 + CTCs	Eribulin anti-PD-1 antibody	2022–23	Up to 10 months	Prognostication	NCT05402722
	NA	Observational	Prospective Cohort study	Monocentric	Real time molecular analysis of CTC dynamics post-neoadjuvant chemotherapy	150	CTC phenotypic characterization (RNA-seq)	NA	NA	2022–30	NA	Therapeutic monitoring	NCT04504747
	II	Interventional	Single arm Open-label	Monocentric	Evaluation of CTC dynamics pre- & post-treatment	20	CTC count	NA	Fulvestrant Anastrozole Abemaciclib	2022–28	Up to 5 years	ORR, PFS, OS Prognostication Therapeutic monitoring	NCT05524584
Cervical cancer	NA	Interventional	Pilot study Open-label Single group	Multicentric	Evaluation of CTC spread at different steps of surgery	20	CTC count	Cut-off: > 1 CTC/7.5 mL	NA	2021–29	3–5 years	DFS Prognostication	NCT04770090
	0	Interventional	Single arm Open-label	Monocentric	Detection of CTCs	30	CTC count	NA	NA	2023–24	3 months post-therapy	Diagnosis	NCT05462951
Colon cancer	NA	Interventional	Randomized controlled trial Parallel study Open-label	Monocentric	Comparison of Open D3 Right Hemicolectomy with Laparoscopic CME wrt. CTCs	126	CTC count	CTC/ml blood	Surgical	2016–26	60 months	Prognostication	NCT03776591
	I	Interventional	Single arm Open-label	Monocentric	Evaluation of PDL1 + CTC dynamics pre- & post-treatment	18	CTC count CTC characterization	PDL1 + CTCs	Magicell-NK contains NK cells	2022–24	Up to 60 weeks	SAE, TEAE, RP2D, MTD, DFS Prognostication Therapeutic monitoring	NCT05394714
Colorectal Cancer	NA	Interventional	Single arm Open-label	Monocentric	Isolation of heterogenous population of CTCs	100	CTC characterization	NA	NA	2016–25	54 months	Diagnosis	NCT04186117
	NA	Interventional	Single arm Open-label	Monocentric	CTCs as a predictor of progression risk & metastasis	120	CTC count CTC characterization	NA	NA	2017–25	4 (± 1) weeks post-surgery	Prognostication	NCT03256084
	NA	Observational	Case–control Prospective	Monocentric	Evaluate relevance of anesthesia in tumor metastasis and recurrence on the basis of CTC dynamics	260	CTC count	NA	Propofol Sevoflurane Remifentanil	2017–23	Up to 5 years	Cancer free survival Cancer recurrence rate Cancer metastasis rate Prognostication Therapeutic monitoring	NCT03193710
	IV	Interventional	Randomized Open-label	Multicentric	Monitoring change in no. of CTCs	120	CTC count	NA	Morphine Piritramid Epidural	2019–23	2019	Therapeutic monitoring	NCT03700411
Ewing Sarcoma Family of Tumors	II	Interventional	Non- randomized controlled Parallel Open-label	Multicentric	Evaluation of CTC dynamics pre- & post-treatment	45	CTC count	NA	VDC-IE × 2 VDC-IE TEMIRI BuMel Surgery Radiotherapy	2016–23	Every 3 months during 2 years	EFS Prognostication Therapeutic monitoring	NCT03011528
Gastric cancer	II	Interventional	Single arm Open-label	Monocentric	Explore clinical value of CTC dynamics	40	CTC count	NA	Neoadjuvant chemotherapy	2019–24	Pre- and post- neoadjuvant chemothe-rapy (2 years)	DFS, OS Prognostication Therapeutic monitoring	NCT03957564
	NA	Observational	Retrospective/Prospective Cohort study	Multicentric	Genetic profiling of CTCs in respondents vs non-respondents	250	CTC genetic characterization	NA	NA	2020–23	36 months	Therapeutic monitoring	NCT04842916
	NA	Observational	Case-only Prospective study	Monocentric	Correlate CTC count with survival	150	CTC count	NA	NA	2021–24	24 months	OS, PFS Disease recurrence Peritoneal seeding Prognostication	NCT05752357
	NA	Observational	Case–control Prospective study	Multicentric	Explore influence of CTCs on metastasis	200	CTC count CTC characterization	NA	NA	2022–24	NA	Prognostication	NCT05208372
	NA	Interventional	Single arm Open-label	Monocentric	Examination of presence of bacteria in CTCs	20	CTC characterization (RNAscope®)	NA	Gastrectomy	2023–25	NA	Identification of intra-tumoral microbiota and its taxonomic characterization	NCT05800236
Gastro-esophageal Cancer	I	Observational	Prospective Cohort study	Monocentric	Detection of CTCs for determination of therapeutic regimen	100	CTC count	CellSearch (≥ 1 CTC / 7.5 mL)	NA	2021–28	NA	Diagnosis Prognostication	NCT04455282
Gastrointestinal cancer	NA	Observational	Prospective Cohort study	Monocentric	Detection of CTCs	1000	CTC count & characterization	NA	NA	2011–24	NA	Diagnosis	NCT01831609
Glioma	NA	Interventional	Non- randomized controlled Parallel Open-label	Monocentric	Evaluation of CTC dynamics pre- & post-surgery	50	CTC count CTC characterization	NA	Neurosurgery	2022–23	2 days/ 3 months/ 14 months	Prognostication Therapeutic monitoring	NCT05133154
Hepatocellular carcinoma	NA	Observational	Case–control Prospective study	Multicentric	Association between CTC and risk of recurrence post-surgery	200	CTC count CTC phenotypic characterization	FACSymphonyEpCAM, N-cadherin, CD90 EMT index (EpCAM + /N-cad- & EpCAM + /N-cad +)	NA	2019–24	5 years	DFS, OS Diagnosis Prognostication	NCT04800497
	II	Interventional	Single arm Open-label	Monocentric	Evaluation of PDL1 + CTC dynamics pre- & post-treatment	37	CTC count CTC characterization	PDL1 + CTCs	Durvalumab Stereotactic body radiation therapy	2020–24	36 months	PFS, ORR, OS, LCR Prognostication Therapeutic monitoring	NCT04913480
	NA	Interventional	Randomized Controlled Double-blind Parallel study	Monocentric	Effects of Mode of Anaesthesia during surgery on CTCs	220	CTC count	NA	Propofol Sevoflurane	2020–24	Pre-operative, Intra-operative, immediately post-operative and 24 h post-surgery	Therapeutic monitoring	NCT04601961
	NA	Observational	Prospective Cohort study	Monocentric	Evaluate prognostic value of CTCs and detect corresponding mutation load	300	CTC count scWGS	Inertial focusing principle-based microfluidic device	NA	2021–24	2 years	PFS Therapeutic monitoring Prognostication	NCT05242237
Head and neck cancer	NA	Interventional	Single arm Open-label	Monocentric	Establish a correlation between TGV, TK, & CTC with reponse	100	CTC count	NA	MRI	2017–23	Up to 2–3 months	Therapeutic monitoring	NCT03491176
	NA	Observational	Prospective Cohort study	Monocentric	Correlation between PDL1 + CTCs & outcome of patients undergoing CCRT	50	CTC characterization	PDL1 + CTCs	NA	2021–22	1 yr	PFS, OS Loco-regional recurrence rate Distant metastasis rate Prognostication Therapeutic monitoring	NCT05008796
Lung cancer	NA	Observational	Prospective Cohort study	Monocentric	Evaluate CTC dynamics post definitive radiotherapy/ chemoradiotherapy	153	CTC count	Adenovirus transduction-based CTC imaging exhibiting high levels of telomerase activity GFP + CTCs /mL	NA	2012–23	24 months	Therapeutic monitoring	NCT02135679
	NA	Interventional	Single arm Open-label	Monocentric	Evaluation of senescence markers in CTCs	10	CTC characterization	NA	Dexamethasone PET	2016–23	Till day 9	Diagnosis	NCT02819024
	NA	Observational	Prospective Cohort study	Monocentric	Variation trend in pre-operation CTC count	65	CTC count	NA	NA	2017–23	5 years	Malignancy prediction capacity Prognostication	NCT03724500
	NA	Observational	Cohort study Case-only Prospective study	Multicentric	Ability of CTC load to predict symptoms, response, and disease recurrence	80	CTCs derived xenograft DNA repair mutations in CTCs	NA	NA	2017–23	Every 6–12 weeks	Diagnosis, prognosis and therapeutic monitoring	NCT02630615
	NA	Observational	Prospective	Monocentric	Establish correlation between PDOs and CTCs	150	CTC count	NA	NA	2018–24	NA	PDOs-CTCs correlation	NCT03655015
	NA	Observational	Prospective Case–control Double arm study	Monocentric	Evaluate CTC dynamics pre- & post-surgery with/without use of ECMO/CPB	20	CTC count	NA	NA	2019–22	24 h after surgery	Therapeutic monitoring	NCT04048512
	NA	Interventional	Single arm Open-label	Monocentric	Identification of mRNA tumor markers by CTC pre- & post-surgery	39	CTC count CTC characterization	NA	Surgical resection SBRT	2020–23	Pre- & Post (6 weeks) surgery	OS, DFS, pCR, Prognostication Therapeutic monitoring	NCT04160143
	NA	Observational	Prospective Cohort study	Monocentric	Detection and capture efficiency of viable CTCs by microfluidic device	40	CTC isolation CTC culture (In vitro & in ovo)	Novel microfluidic device (LUTON) CTC enrichment (ClearCellFX)	NA	2021–23	NA	Diagnosis	NCT04957602
Melanoma	NA	Observational	Case–control Prospective	Monocentric	In vivo real-time detection of CTCs	75	CTC detection	Photoacoustic flow cytometry (PAFC)	NA	2013–23	NA	Diagnosis	NCT01776905
Nasopharyngeal carcinoma	NA	Observational	Case-only Prospective	Monocentric	Evaluation of chemosensitivity and treatment efficacy via CTC dynamics	50	CTC count	NA	Cisplatin-based chemotherapy	2020–25	1 year	PFS Prognostication Therapeutic monitoring	NCT04544969
	II	Interventional	Randomized controlled Parallel Open-label	Monocentric	Evaluation of PDL1 + CTC dynamics pre- & post-treatment	118	CTC characterization	PDL1 + CTCs	Durvalumab Cisplatin Gemcitabine	2020–24	36 months	PFS, OS, ORR Prognostication Therapeutic monitoring	NCT04447612
Neuroblastoma	II	Interventional	Single arm Open-label	Multicentric	Analysis of CTC dynamics pre- & post-treatment	140	CTC analysis	NA	DFMO	2015–23	5 years	EFS, OS Prognostication Therapeutic monitoring	NCT02395666
Neuroendocrine tumors	I/II	Interventional	Single arm Open-label	Multicentric	Evaluation of CTC response pre- & post-treatment	49	CTC characterization	FACS	Cabozantinib Lanreotide	2021–25	72 months	Therapeutic monitoring	NCT05048901
Oropharyngeal Squamous Cell Carcinoma	NA	Observational	Prospective Cohort study	Monocentric	To evaluate prognostic value of CTCs	40	CTC count & characterization Transcriptomic profiling (scRNAseq)	ClearCell® FX CK, CD44, N-cadherin and PD-L1	NA	2021–27	NA	Prognostication	NCT04696744
Pancreatic cancer	II	Observational	Prospective Cohort study	Multicentric	Predict therapeutic regimens according to CTC status	74	CTC isolation CTC PGx analysis	Proprietary invasion assay	NA	2017–24	36 months	PFS Diagnostic Prognostication Therapeutic monitoring	NCT03033927
	NA	Interventional	Randomized controlled Parallel study Open-label	Monocentric	Correlation of CTC count during pancreatico- duodenectomy with local recurrence, metastasis and survival	86	CTC count	CTCs/ mL blood	NA	2017–23	NA	Prognostication	NCT03340844
	I (Early)	Interventional	Prospective Randomized controlled Double blinded Placebo-controlled	Monocentric	Determine effect of lidocaine infusion on CTC dynamics	46	CTC count Src Tyrosine Kinase Enzymatic activity in CTCs	NA	Lidocaine Hydrochloride	2018–23	NA	Prognostication Therapeutic monitoring	NCT04048278
	NA	Interventional	Single arm Open-label Prospective	Monocentric	Evaluation of levels of CTCs/mL to evaluate effect of EUS-FNA on tumor progression	42	CTC count CTC characterization	> 4 CTCs/ml	NA	2021–25	NA	Inference of procedural impact from CTCs	NCT04677244
	NA	Interventional	Single arm Open-label	Monocentric	Evaluation of feasibility of EUS-guided Portal Circulation sampling for isolation, enumeration and profiling of CTCs	70	CTC count & characterization	Microfluidic enrichment of CTCs per 7.5 mL blood	NA	2022–27	NA	Diagnosis	NCT05247164
	NA	Interventional	Single arm Open-label	Monocentric	Concordance between 2 CTC isolation techniques	63	CTC count CTC culture	CellSearch EPIDROP (RosetteSep enrichment + microfluidic chip based) CTC-AXL( +)	NA	2022–24	36 months	OS, PFS, SV, SP Prognostication	NCT05346536
Pediatric cancer	IV	Interventional	Single-blinded Randomized controlled Parallel study	Monocentric	Effects of Mode of Anaesthesia during surgery on CTCs	100	CTC count	CTCs/100 µL	Propofol Sevoflurane	2021–28	Intraoperative to postoperative 24 h	Therapeutic monitoring	NCT04475705
Prostate cancer	I	Interventional	Single arm Open-label	Monocentric	Evaluation of CTCs post-surgery	1045	CTC count	NA	Prostatic surgery	2007–23	NA	Therapeutic monitoring	NCT00977457
	I	Interventional	Single arm Open-label	Monocentric	Evaluation of CTC dynamics pre- & post-treatment	13	CTC count	NA	Engineered autologous T cells Cyclo-phosphamide	2010–23	Weeks 4, 12, 24 and every 3 months	Therapeutic monitoring	NCT01140373
	NA	Observational	Prospective Cohort study	Multicentric	Evaluation of CTC dynamics pre- & post-therapy	92	CTC count	NA	CYP-17 Inhibition Therapy	2013–23	12–14 weeks	PFS Prognostication	NCT01953640
	Ib/II	Interventional	Randomized controlled Parallel study Open-label	Multicentric	Determine anti-tumor activity of treatment based on CTCs	120	CTC count	< 5 CTCs/7.5 ml blood	BI 836845 Enzalutamide	2014–23	Up to 3 years	Therapeutic monitoring	NCT02204072
	II	Interventional	Single arm Open-label	Multicentric	Correlation between CTC count and PSA expression	36	CTC count	NA	Enzalutamide	2014–23	Up to 5 years	Prognostication Therapeutic monitoring	NCT02099864
	II	Interventional	Randomized Parallel study Double-blind Placebo controlled	Multicentric	Evaluation of CTC dynamics pre- & post-treatment	158	CTC count	NA	Olaparib Abiraterone Prednisone or prednisolone	2014–23	~ 3 years	PFS, OS Prognostication Therapeutic monitoring	NCT01972217
	NA	Interventional	Randomized controlled Parallel Open-label	Monocentric	Evaluation of CTC dynamics pre- & post-therapy	164	CTC count	NA	Radiotherapy	2015–29	Up to 2 years	ORR, pCR Prognostication Therapeutic monitoring	NCT02307058
	I	Interventional	Single arm Open-label	Multicentric	Evaluation of CTC dynamics pre- & post-treatment	16	CTC count	CellSearch	Apalutamide Abiraterone acetate Docetaxel Prednisone	2016–23	Up to 100 months	PFS Prognostication Therapeutic monitoring	NCT02913196
	II	Interventional	Single arm Open-label	Multicentric	Evaluation of CTC dynamics pre- & post-treatment	289	CTC count	NA	Niraparib	2016–23	At 8 weeks, Up to 52 months	OS, rPFS Prognostication Therapeutic monitoring	NCT02854436
	II	Interventional	Randomized controlled Parallel study Open-label	Multicentric	Determination of therapeutic regimen	196	CTC characterization	AR-specific CTCs	Abiraterone Acetate Apalutamide Cabazitaxel Carboplatin Ipilimumab Prednisone	2016–23	Up to 4 years	OS Prognostication Therapeutic monitoring	NCT02703623
	NA	Observational	Prospective Cohort study	Monocentric	Evaluate CTC dynamics pre- and post-treatment	144	CTC count CTC culture	NA	NA	2016–31	2–2.5 years post-treatment	Therapeutic monitoring Prognostication	NCT02997709
	I/II	Interventional	Non- randomized controlled Parallel study Open-label	Multicentric	Evaluation of CTC dynamics pre- & post-treatment	86	CTC count	< 5/7.5 ml	AZD5069 Enzalutamide 40 MG	2017–23	12–24 months	OS Prognostication Therapeutic monitoring	NCT03177187
	I	Interventional	Single arm Open-label	Monocentric	Evaluation of CTC response pre- & post-treatment	32	CTC count	CellSearch	225Ac-J591	2017–24	After 12 weeks	Therapeutic monitoring	NCT03276572
	I/II	Interventional	Non- randomized controlled Parallel Open-label	Monocentric	Evaluation of CTC dynamics pre- & post-treatment	29	CTC count	NA	PROSTVAC-V/F Nivolumab	2017–23	3–5 years	Therapeutic monitoring	NCT02933255
	II	Interventional	Single arm Open-label	Multicentric	Evaluation of CTC dynamics pre- & post-treatment and correlation with survival	128	CTC count	> 1 CTC/7.5 mL of Blood	Talazoparib	2017–24	~ 56 months	OS, PFS Prognostication Therapeutic monitoring	NCT03148795
	II	Interventional	Single arm study Open-label	Multicentric	Therapeutic implication of ARV7 + CTC count	140	CTC count	ARV7 + CTCs ≥ 5 CTCs per 7.5 mL blood	Cabazitaxel Antihistamine Corticosteroid H2 antagonist Antiemetic	2017–22	9- 12 weeks	PFS, OS PSA response Prognostication Therapeutic monitoring	NCT03050866
	I/II	Interventional	Non- randomized controlled Sequential Open-label	Multicentric	Evaluation of CTC dynamics pre- & post-treatment	136	CTC count	< 5 per 7.5 mL	Niraparib Cetrelimab Cetrelimab Abiraterone acetate Prednisone	2018–23	Up to 42 months	ORR, OS Prognostication Therapeutic monitoring	NCT03431350
	II	Interventional	Single arm Open-label	Monocentric	Correlate ARV7 + CTCs with PSA response	30	CTC characterization	ARV7 + CTCs	Docetaxel Enzalutamide	2019–23	6/12/24 months	Prognostication Therapeutic monitoring	NCT03700099
	Ib/II	Interventional	Randomized controlled Parallel study Open-label	Multicentric	Evaluation of CTC dynamics pre- & post-treatment	240	CTC count	Veridex assay (< 5/7.5 mL)	BXCL701 plus Pembrolizumab BXCL701 monotherapy	2019–25	Up to 36 months	Therapeutic monitoring	NCT03910660
	I/II	Interventional	Non- randomized controlled Parallel Open-label	Multicentric	Evaluation of CTC dynamics pre- & post-treatment	216	CTC count	< 5 cells/7.5 mL	REGN5678 Cemiplimab 18F-DCFPyL Sarilumab	2019–25	Up to 5 years	ORR Prognostication Therapeutic monitoring	NCT03972657
	I	Interventional	Non- randomized controlled Sequential Open-label	Multicentric	Evaluation of CTC dynamics pre- & post-treatment	90	CTC count	< 5 cells/7.5 mL	ORIC-101 Enzalutamide [Xtandi]	2019–23	36 months	RP2D, PSA response rate, DOR, ORR, PFS, OS Prognostication Therapeutic monitoring	NCT04033328
	Ib/II	Interventional	Single arm Open-label	Monocentric	Evaluation of CTC dynamics pre- & post-treatment	10	CTC count	< 5 cells/7.5 mL	Enzalutamide Venetoclax	2019–23	Up to 3 years	DOR, ORR, OS, PFS Prognostication Therapeutic monitoring	NCT03751436
	I/II	Interventional	Non- randomized controlled Parallel Open-label	Multicentric	Evaluation of CTC response pre- & post-treatment	55	CTC count	< 5/7.5 ml	Abiraterone Acetate Tildrakizumab	2020–24	12 months	Therapeutic monitoring	NCT04458311
	I	Interventional	Sequential Open-label	Multicentric	Evaluation of CTC dynamics pre- & post-treatment	39	CTC count	NA	Cyclophosphamide (Non-IMP) Fludarabine (Non-IMP) UniCAR02-T-pPSMA UniCAR02-T (IMP)	2020–24	12 months	Therapeutic monitoring	NCT04633148
	I	Interventional	Non- randomized controlled Sequential Open-label	Multicentric	Evaluation of CTC dynamics pre- & post-treatment and correlation with survival	464	CTC count	NA	AMG 509 Abiraterone Enzalutamide Docetaxel	2020–28	Up to 3 years	DOR, ORR, PSA response PFS, OS Prognostication Therapeutic monitoring	NCT04221542
	I/II	Interventional	Non- randomized controlled Parallel Open-label	Multicentric	Evaluation of CTC dynamics pre- & post-treatment	105	CTC count	CellSearch	225Ac-J591 68 Ga-PSMA-HBED-CC injection	2020–27	Up to 100 months	OS Prognostication Therapeutic monitoring	NCT04506567
	II	Interventional	Randomized controlled Parallel study Open-label	Monocentric	Correlation of CTCs with tumor burden	120	CTC count	NA	Cabazitaxel Carboplatin Cetrelimab Niraparib	2020–25	Up to 5 years	PFS, OS Prognostication Therapeutic monitoring	NCT04592237
	I (Early)	Interventional	Single arm Open-label	Multicentric	Evaluation of CTC dynamics pre- & post-treatment	18	CTC count	CellSearch	225Ac-J591	2020–24	Up to 100 months	OS, PFS Prognostication Therapeutic monitoring	NCT04576871
	NA	Interventional	Non-randomized Parallel study Open-label	Multicentric	Detection of CTCs for early diagnosis	320	CTC count	EPIDROP	NA	2021–33	NA	Diagnosis	NCT04556916
	NA	Interventional	Single arm Open-label	Multicentric	Capture viable CTCs	100	CTC isolation	EPIDROP (10 mL) EpCAM, PSMA, CD45 CellSearch	NA	2021–23	18 months	SV, SP Diagnosis	NCT04581109
	I	Interventional	Open-label Non-randomized	Monocentric	Determine anti-tumor activity of treatment based on CTCs	42	CTC count	≤ 4 cells/7.5 mL	IMU-935	2021–23	6 months	Therapeutic monitoring	NCT05124795
	II	Interventional	Single arm Open-label	Multicentric	Evaluation of CTC dynamics pre- & post-treatment	30	CTC count	CellSearch	Cabozantinib	2021–25	100 months	OS Prognostication Therapeutic monitoring	NCT04631744
	I/II	Interventional	Single arm Open-label	Multicentric	Evaluation of CTC dynamics pre- & post-treatment	33	CTC count	CellSearch	225Ac-J591 177Lu-PSMA-I&T 68 Ga-PSMA-11	2021–27	Up to 100 months	PFS, rRR Prognostication Therapeutic monitoring	NCT04886986
	I/II	Interventional	Randomized controlled Sequential Open-label	Multicentric	Evaluation of CTC dynamics pre- & post-treatment	136	CTC count	NA	Acapatamab Enzalutamide Abiraterone AMG 404	2021–23	Up to 3 years	PFS, OS Prognostication Therapeutic monitoring	NCT04631601
	III	Interventional	Randomized controlled Parallel Open-label	Multicentric	Evaluation of CTC dynamics till progression	114	CTC count	NA	Stereotactic body radiotherapy Androgen deprivation therapy Radiotherapy	2021–29	Till progress-ion	Prognostication	NCT04983095
	NA	Observational	Prospective Cohort study	Multicentric	Characterization of CTCs	100	CTC count CTC genotypic and phenotypic characterization	Parsortix® System	NA	2022–25	NA	PFS, DFS, OS Prognostication Therapeutic monitoring	NCT05437679
	NA	Observational	Double-blinded Prospective Paired cohort study	Monocentric	Value of CTC positivity in predicting post-RP treatment failure	490	CTC count	NA	NA	2022–27	10 (3 months interval for 1st yr followed by yearly)	MFS, OS Treatment stratification Prognostication	NCT05533515
	II	Interventional	Single arm Open-label	Monocentric	Evaluate CTC dynamics pre- & post-treatment	34	CTC count	< 5 CTCs/7.5 mL	Enfortumab Vedotin	2022–26	12 months	Therapeutic monitoring	NCT04754191
	I	Interventional	Sequential Open-label	Monocentric	Evaluation of CTC dynamics pre- & post-treatment	20	CTC count	NA	CART-PSMA cells	2023–25	Up to 2 years	Therapeutic monitoring	NCT05656573
	II	Interventional	Single arm Open-label	Monocentric	Evaluation of CTC dynamics pre- & post-treatment	50	CTC count	< 5 CTCs/7.5 mL	Cabozantinib Nivolumab	2023–24	6 months – 3 years	rPFS, PSA response, OS, ORR Prognostication Therapeutic monitoring	NCT05502315
Rectal cancer	NA	Observational	Prospective Cohort study	Monocentric	Change in CTC dynamics pre- and post-neoadjuvant therapy	520	CTC count	NA	NA	2013–23	NA	Prognostication Therapeutic monitoring	NCT02874885
	NA	Observational	Prospective Cohort study	Monocentric	Compare CTC dynamics during different time points of surgery	100	CTC count	NA	Surgical approach	2021–25	3 years	DFS Prognostication	NCT05109130
	NA	Observational	Prospective Cohort study	Monocentric	Development of response prediction model based on CTCs	50	CTC count	NA	NA	2022–30	5 years	Prognostication Tumor regression grading	NCT05524012
Urinary bladder carcinoma	NA	Interventional	Randomized controlled trial Parallel study	Multicentric	Evaluate CTC dynamics pre- & post-surgery (TURBT & PKVBT)	40	CTC count	Automated immunofluorescence Microscopy (15 mL) (CK20 + , p53 + , DAPI + & CD45-)	NA	2021–25	Pre- & post-surgery	Tumor recurrence Prognostication Therapeutic monitoring	NCT04811846
Uterine cancer	NA	Observational	Case-only Prospective	Monocentric	Determination of CTC count and their genomic alterations	580	CTC count CTC characterization	NA	NA	2012–24	12 months	Diagnosis	NCT03624712
Adrenal Gland Pheochromocytoma Hematopoietic and Lymphoid System Neoplasm Malignant Solid Neoplasm Paraganglioma	II	Interventional	Single arm Open-label	Multicentric	Explore genetic alterations in CTCs	34	CTC characterization (EMT index & genomic alterations)	NA	Computed Tomography Talazoparib Temozolomide	2022–26	Up to 3 years	ORR, PFS Prognostication Therapeutic monitoring	NCT05142241
Breast cancer Prostate cancer Ovarian cancer	NA	Interventional	Prospective interventional Single arm study Open-label	Monocentric	Identification of CTCs	76	CTC phenotypic characterization	New isolation technology HER2 + (FISH) CA125 + (IHC) PSA + (IHC)	NA	2020–23	NA	Feasibility of CTC isolation	NCT03979339
Cancer	NA	Interventional	Non- randomized controlled Parallel study Open-label	Multicentric	Establishment of CDX models	760	CDX models	NA	NA	2015–24	18 months	Diagnostic	NCT02866149
	NA	Observational	Cohort study	Multicentric	Investigate the role & biology of CTCs and CTC-clusters during disease progression	3000	CTC count CTC culture	NA	NA	2020–30	Up to 240 months	OS, PFS Prognostication	NCT04520672
Cell cancer	NA	Observational	Case–control	Monocentric	Establishment of 3D cell culture model of CTCs	120	CTC isolation/ purification/ proliferation	Label-free CTCs	NA	2021–24	NA	Diagnosis	NCT05623748
Colorectal cancer Metastatic cancer	NA	Observational	Prospective Cohort study	Monocentric	Predict therapeutic regimens according to CTC status	60	CTC count	ANGLE Parsortix™ system	NA	2023	NA	Prognostication	NCT05648240
Colorectal Carcinoma Lung Carcinoma Pancreatic Carcinoma	II	Interventional	Single arm Open-label	Monocentric	Evaluation of CTC dynamics pre- & post-treatment and their correlation with PDL1	53	CTC characterization	PDL1 + CTCs	Danvatirsen Durvalumab	2017–24	Up to 4 years	DCR, ORR, OS, PFS Prognostication Therapeutic monitoring	NCT02983578
Hepatocarcinoma Pancreatic Tumor	NA	Interventional	Non- randomized controlled Parallel Open-label	Monocentric	Identification of CTCs on the basis of their metabolomic profile and their correlation with prognosis	300	CTC characterization	NA	NA	2022–26	24/36 months	Diagnosis Prognostication	NCT05794048
Hepatocellular Carcinoma Colorectal Cancer Gastric Cancer Pancreatic Cancer	I	Interventional	Sequential Open-label	Monocentric	Evaluate the clearance effect of CAR-T cell treatment on CTCs	48	CTC count	NA	EPCAM CAR-T	2021–24	Up to 3 years	PFS, DOR, OS, DCR, ORR, TEAE Prognostication Therapeutic monitoring	NCT05028933
Melanoma Sarcoma SCC Pancreatic Cancer Prostate Cancer Breast Cancer	NA	Observational	Prospective Cohort study	Monocentric	Isolate and quantify CTCs using HSP70	120	CTC count	HSP70 + vs EpCAM + CTCs	NA	2021–22	3 months	PFS, OS Prognostication	NCT04628806
Neoplasms Myelodysplastic Syndromes Lymphomas	I	Interventional	Single arm Open-label	Monocentric	Evaluation of CTC apoptosis post-therapy	75	CTC characterization	NA	Bortezomib Clofarabine	2014–23	Cyclic (21 days)	Therapeutic monitoring	NCT02211755
Non-small Cell Lung Cancer Prostate Cancer Renal Cancer Transitional Cell Carcinoma	I	Interventional	Sequential Open-label	Multicentric	Evaluation of CTC dynamics pre- & post-treatment	24	CTC count	NA	GEM3PSCA	2019–23	Day 8 / EOT	Therapeutic monitoring	NCT03927573
Non-Small Cell Lung Cancer Colorectal Cancer Cutaneous Squamous Cell Carcinoma Hepatocellular Carcinoma Castration-resistant Prostate Cancer Ovarian Cancer	II	Interventional	Single arm Open-label	Multicentric	Evaluation of CTC dynamics pre- & post-treatment	320	CTC count	NA	SOT101 Pembrolizumab	2022–25	~ 2 years	ORR, DOR, CBR, PFS, TtR, PSA response Prognostication Therapeutic monitoring	NCT05256381
Prostate Cancer Breast Cancer Lung Cancer Ovarian Cancer Gastric Cancer Colorectal cancer	NA	Interventional	Single arm Open-label	Multicentric	Evaluate automated system to culture metastatic CTCs in embryonated chicken eggs	132	CTC Ovo-culture (PDX)	NA	NA	2023–24	19 days	PDX development	NCT05472532
Solid tumors	I	Interventional	Single arm Open-label	Monocentric	Determine effect of treatment on re-expression of select genes silenced by methylation in CTCs	50	CTC characterization	NA	aza-TdC	2018–24	Cyclic (21 days)	Therapeutic monitoring	NCT03366116
	II	Interventional	Single arm Open-label	Multicentric	Evaluation of CTC dynamics pre- & post-treatment	202	CTC count	NA	Avelumab Talazoparib	2018–23	~ 24 months	OR, TtR, DOR, OS, PFS, PSA response Prognostication Therapeutic monitoring	NCT03565991
	II	Interventional	Non- randomized controlled Single arm Open-label	Multicentric	Evaluation of CTC dynamics pre- & post-treatment	61	CTC count	< 5/7.5 mL blood	Ceralasertib	2020–23	~ 3 years	Therapeutic monitoring	NCT04564027
Tobacco-related cancers	NA	Interventional	Prospective Non-randomized Open-label Single arm Cohort study	Monocentric	Evaluate screening value of CTCs	176	CTC identification	NA	NA	2017–23	NA	Diagnostic value	NCT02849041

*AR* androgen receptor, *ARV7* androgen receptor splice variant, *BOR* best overall response, *CAR-T* chimeric antigen receptor T-cell therapy, *CBR* clinical benefit rate, *CD45* cluster of differentiation 45, *CDX* CTC-derived xenografts, *CK* cytokeratin, *CTC* circulating tumor cell, *DAPI* 4’,6-diamidino-2-phenylindole, *DCR* disease control rate, *DFS* disease free survival, *DOR* duration of response, *ECMO/CPB* extracorporeal membrane oxygenation/cardiopulmonary bypass, *EFS* event free survival, *EMT* epithelial mesenchymal transition, *EOT* end of treatment, *EpCAM* epithelial cell adhesion molecule, *ER* estrogen receptor, *EUS-FNA* endoscopic ultrasound-guided fine-needle aspiration biopsy, *FR* folate receptor, *HER2* human epidermal growth factor receptor 2, *HSP70* heat shock protein 70, *iDFS* invasive disease-free survival, *IHC* immunohistochemistry, *ISH* in-situ hybridization, *LCR* local control rate, *MFS* metastasis free survival, *MRI* magnetic resonance imaging, *MTD* maximum tolerated dose, *NA* not applicable, *ORR* objective response rate, *OS* overall survival, *pCR* pathological complete response, *PDL1* programmed death ligand 1, *PDO* patient-derived organoids, *PDX* patient-derived xenografts, *PET* positron emission tomography, *PFS* progression free survival, *PGx* pharmacogenomics, *PSA* prostate specific antigen, *PSMA* prostate-specific membrane antigen, *RFS* relapse free survival, *RP* radical prostatectomy, *RP2D* recommended Phase II dose, *rPFS* radiographic progression free survival, *rRR* radiographic response rate, *SAE* serious adverse events, *SBRT* stereotactic body radiation therapy, *scWGS* single-cell whole genome sequencing, *SP* specificity, *SV* sensitivity, *TEAE* treatment emergent adverse events, *TGV* tumor growth velocity, *TK* tumor kinetics, *TTNM* time to new metastases, *TtR* time to response, *TURBT* transurethral resection of bladder tumor

### CTC as a diagnostic marker

CTC detection presents an appealing minimally-invasive approach for aiding cancer diagnosis. By the time a tumor lesion becomes detectable through conventional imaging techniques, it has typically already progressed significantly. CTCs have been proposed to facilitate early detection of cancers [80], particularly those originating from deep-seated tumors, effectively bridging the gap between tumor progression and late diagnosis, especially in rapidly advancing cancers. Table 3 summarizes studies that support CTCs can effectively serve as diagnostic markers. Detection of EpCAM^+^ CTCs using fluorescence-activated cell sorting (FACS) in gastric cancer showed high sensitivity (SV) and specificity (SP) of 92.3% and 100%, respectively [81]. Whereas magnetic NPs showed reduced SV of 49.1% [82]. CTC detection in CaCx using immunomagnetic bead negative enrichment technique showed moderate SV (84.13%) with high SP (93.65%) [83]. Identification of gliomas using a cutoff of ≥ 2 CTCs/mL by subtraction enrichment and immunostaining-fluorescence in situ hybridization (SE-iFISH) demonstrated high diagnostic SV and SP of 100 and 91.2%, respectively [84]. Detection of CTCs in lung cancer exhibited variable SV ranging from 52.94 to 85.85% and SP ranging from 78.57 to 100% using various techniques [52, 85–88]. Detection of folate receptor (FR)^+^ CTCs in prostate cancer using CytoploRare Kit has showed high SV (100%) but compromised SP (76.7%) [89]. Further, detection of ≥ 1 CTC based on epithelial markers such as EpCAM and CK in HCC revealed a moderate diagnostic performance [90, 91]. In pancreatic cancer, using a cutoff of 2 CTCs/3.2 mL of blood resulted a SV of 76% and SP of 94% [92]. The detection of CTC by negative enrichment using NanoVelcro system in tongue squamous cell carcinoma exhibited moderate SV (83%) and SP (75%) [53]. Assessment of diagnostic performance of CTCs in CRC studies revealed high SP of 100% using isolation strategies such as centrifugal microfluidic system and negative enrichment immunofluorescence-in situ hybridization (NE-imFISH) [93–97]. Using chromosome enumeration probe 7/8 (CEP7/8) panel for identification of CTCs in ESCC demonstrated a SP of 96.74% and SV of 70.54% [98]. High diagnostic SP and SV of 98% and 94.32%, respectively was observed in oral squamous cell carcinoma (OSCC) [99]. CTCs isolated from breast cancer depicted a high SP ranging from 93.75 to 100% and moderate to high SV (50–80%) [54, 100, 101]. Meta-analyses of CTC-detection studies in thyroid cancer [102], breast cancer [103], and lung cancer [104] resulted in a low to moderate SVs (50–79%) with moderate to high specificity of (84%—93%). Utilizing panel of carbohydrate antigen 19–9 (CA19-9), Maspin, EpCAM, and CK for CTC detection in pancreaticobiliary cancers registered both a high SV (95.9–96.4%) and SP (92.3–100%) using CTC enrichment medium and immunocytochemistry (ICC) [105].

**Table 3:** Circulating tumor cells as a diagnostic marker for different types of cancer

Type of cancer	Sample size (n)	CTC enrichment technique	Nature of markers used for CTC detection	CTC range	Sensitivity (%)	Specificity (%)	References
Gastric Cancer	26	FACS	EpCAM + CD44 +	69.9 ± 52.0	92.3	100	[81]
	92	Magnetic NPs	EpCAM +	NA	49.1	95	[82]
Cervical cancer	63	Immunomagnetic bead negative enrichment technique	NA	0–3/ 4 mL blood	84.13	93.65	[83]
Gliomas	22	SE-iFISH	DAPI + , CD45-, and heteroploidy CEP8	> 2 CTCs	100	91.2	[84]
Lung Cancer	64	GILUPI CellCollector	EpCAM, CD45, CK7/19/panCK, PD-L1	≥ 1 CTC	52.94	90	[52]
	106	CanPatrol™ technique & CytoploRare technique	CK 8, 18, and 19, EpCAM, Vimentin, and Twist, FR +	> 1 CTCs	85.85	78.57	[106]
	108	CytoSorter® mesenchymal CTC kit	CSV, CD45 and DAPI	2 CTCs/ 7.5 mL	67	87	[85]
	18	CEP8 CTC detection kit	DAPI + /CD45 − /CEP8 +	CTC count ≥ 2	75	100	[86]
	380	Cytoplorare® kit	FR + CTC	Cutoff: 9.22 FU/3 ml	69.17–75.20	82.46–83	[87]
	538	Negative enrichment (anti-CD 45 and anti-CD14 immunomagnetic beads) & FR + Cell Detection Kit	FR + CTC	8.9 FU/3 mL	81.91	80.86	[107]
	45	Microfluidic chip with automatic processing system (AF-RCF04)	DAPI + /CD45 − /CK +	11.18	81.8	90.5	[88]
	Meta-analysis (11 studies: n = 3469)	NA	FR + CTC	NA	79	84	[104]
Prostate cancer	30	CytoploRare Kit	FR + CTCs	8.25	100	76.7	[89]
Hepatocellular carcinoma	143	CellSearch™	EpCAM based	CTCs ≥ 1	81.8	72.5	[90]
	145	FACS	CK18/19	Cut-off of 4 ± 1 CTCs/7.5 mL	79–82	81–83	[91]
Pancreatic cancer	168	NE-imFISH	CD45-/DAPI + /CEP8 ≥ 3	2 CTCs/3.2 ml	76	94	[92]
Tongue squamous cell carcinoma	50	NanoVelcro system	DAPI + /CK-/CD45 +	Count of CTC ≥ 4	83	75	[53]
Colorectal cancer	119	Centrifugal microfluidic system with FAST	EpCAM + /CK + , CD45 − , and DAPI +	> 5/7.5 mL	75	100	[93]
	356	CytoploRare® Circulating CRC cell kit (Negative enrichment) & FR-targeted fluorescence quantitative PCR	FR + CTC & HSP90	Cutoff: 11.75 FU	70.55	92.66	[94]
	282	NE-imFISH	CEP8 + /DAPI + /CD45 −	≥ 2 CTCs/3.2 ml	77.78	100	[95]
	238	CytoploRare® Detection Kit	FR + -CTC	9.66 FU/3 ml	61.8	82.6	[96]
	80	Easysep™	CD44	> 1.5 cells/mL	50	88.89	[97]
Pancreatic ductal adenocarcinoma (PDAC)	52	CD-PRIME™ system	EpCAM, CK, Plectin-1 and CD45	0 to 641 cells/3 ml	84.62	96.67	[108]
Esophageal cancer	221	NE-FISH	DAPI + /CD45-/CEP7/8	2/ 3.2 ml	70.54	96.74	[98]
Thyroid cancer (TC)	Meta-analysis (n = 7 studies)	NA	EpCAM/ TSHR	NA	71–78	88–89	[102]
Oral squamous cell carcinoma	152	OncoDiscover	EpCAM +	> 3.5 CTCs	94.32	98	[99]
Breast Cancer	110	FACS	Epithelial, Mesenchymal, CD45	NA	80	100	[54]
	52	Pep@MNPs & ICC	CTC-HER2 +	Cutoff: ≥ 3 CTCs	69.2	100	[100]
	60	CytoploRare® kit	FR + CTC	9.08 Folate Unit/3 ml	63.33	93.75	[101]
	Meta-analysis (n = 16 studies)	NA	NA	NA	50	93	[103]
Pancreaticobiliary Cancers	448	CTC enrichment medium & ICC	CA19.9, Maspin, EpCAM, CK, CD45	NA	95.9–96.4	92.3–100	[105]

*CD45* Lymphocyte common antigen, *CEP8* chromosome enumeration probe 8, *CK* Cytokeratin, *CSV* cell-surface vimentin, *CTC* circulating tumor cell, *DAPI* 4’,6-diamidino-2-phenylindole, *EpCAM* epithelial cellular adhesion molecule, *FACS* fluorescence-activated cell sorting, *FR* folate receptor, *HSP90* heat shock protein 90, *NA* not available, *NE-imFISH* negative enrichment immunofluorescence-in situ hybridization, *PD-L1* programmed cell death ligand 1, *Pep@MNPs* peptide functionalized magnetic nanoparticles, *SE-iFISH* subtraction enrichment and immunostaining-FISH, *TSHR*: thyroid stimulating hormone receptor

From the studies mentioned, EpCAM emerged as the most commonly used marker, both individually and in combination with others like CK^+^, CD45^−^,CD44^+^, and thyroid stimulating hormone receptor (TSHR) in various detection techniques, yielding variable specificity and sensitivity ranges. Utilizing EpCAM, CTC counts ranging from 0 to 641 have been detected in peripheral blood volumes as low as 3–7.5 mL. CTC cutoff ranges in blood are crucial for cancer diagnosis and prognosis, with various markers impacting detection sensitivity and specificity. For instance, using the FACS technique, a cutoff of ≥ 3 CTCs EpCAM^+^ indicated gastric cancer with 100% specificity, demonstrating the importance of marker selection [81]. Similarly, in CRC, a cutoff of > 5 CTCs/7.5 mL detected by a centrifugal microfluidic system using the EpCAM marker also showed 100% specificity [84], underscoring the significance of effective enrichment and detection techniques.

Based on these studies, CTCs can be detected early before the primary tumor is visible using common imaging techniques. While various studies demonstrate high specificity and, in some cases, impressive sensitivity across multiple cancer types as discussed, the overall diagnostic utility of CTCs remains constrained by their rarity in peripheral blood and the variability in sensitivity of the detection technologies. Techniques such as FACS, NE-imFISH, CEP8 CTC detection kit, centrifugal microfluidic systems, and peptide functionalized magnetic nanoparticles (Pep@MNPs) offer high specificity of 100%, but their sensitivity often fluctuates depending on the tumor type, detection marker, and cut-off used. EpCAM stands out as a widely used marker, yet its variable performance across cancers underscores the need for multiparametric approaches. Despite these advances, the variable sensitivity and dependence on sophisticated enrichment platforms limit the routine clinical application of CTCs for routine screening and early detection. Thus, overcoming technical and biological limitations remains critical for their broader implementation in early detection.

### CTC as a prognostic marker

Measuring the number of CTCs has been shown to be an effective method for predicting disease aggressiveness and assessing the response to treatment [109]. Recent reports highlighting the prognostic significance of CTCs have been enlisted in Table 4. The studies consistently showed that higher counts of CTCs are associated with worse clinical outcomes across a range of cancer types. CTC detection itself proved effective in identifying poor prognostic groups within various cancer grades. For instance, in neuroendocrine cancer, a threshold of > 1 EpCAM + CTC/7.5 mL of blood correlated with increased tumor burden [110]. In addition, serial CTC levels are considered as independent prognostic factor for OS and PFS as reported in CRC [111], lung cancer [112], prostate cancer [113], and ESCC [114]. A detection cut-off of ≥ 1 EpCAM^+^CK^+^CTC/8 mL allowed patient stratification based on risk status within different stages of CRC [115].

**Table 4:** Circulating tumor cells as a prognostic marker for different types of cancer

Type of cancer	Sample size (n)	CTC enrichment technique	Nature of markers used for CTC detection	CTC cutoff (CTC count per mL of PB)	Prognostic significance	Refs.
Neuroendocrine cancer	176	Immunomagnetic separation	EpCAM	≥ 1 CTC/ 7.5 mL	↑ CTCs: ↑ tumor burden, ↑ tumor grade, ↑ serum CgA Indicative of worse PFS & OS Within grades, presence of CTCs was able to define a poor prognostic subgroup	[110]
Colorectal Cancer (CRC)	36 studies (n = 3094)	-	-	-	Indicates poor prognosis in primary CRC patients	[116]
	472	CellSearch™	EpCAM	≥ 1 CTC/ 7.5 mL	Not associated with worse DFS and OS No clinicopathological characteristics were significantly associated with the presence of CTC	[117]
	158	CMx platform	CK20 + , DAPI + , CD45-	≥ 5 CTCs/ 2 mL	Eight times more likely to develop distant metastasis within 1 year after curable surgery CTC counts show good correlation with colorectal neoplasm	[118]
	100	FACS	CK19 + /CD45-	≥ 4 CTCs /7 mL	↓ CTCs: ↑ PFS and OS rates during the course of treatment A change in serial CTC levels is considered an independent prognostic factor for OS & PFS	[111]
	118	CellSearch™	EpCAM, CD54-, CK8/18/19 +	≥ 1 CTCs/ 7.5 mL	Associated with advanced age, a high PLR value, and a high NLR value CTC counts were significantly associated with PFS	[119]
	101	CanPatrol™ system	EPCAM, CK8/18/19, VIM, Twist, CD45-	≥ 3 Total CTCs /5 mL	↑ Total and M-CTCs after chemotherapy are significantly related with poor PFS	[120]
	20	CellSearch™ and DEPArray™	EpCAM, CK 8, 18 and CD45-	3 CTCs / 7.5 ml blood	Presence of CTCs at baseline was confirmed a negative prognostic marker	[121]
	74	CanPatrol™ technique	EpCAM, Vimentin	M-CTCs > 1/ 7.5 mL	The positive rate of M-CTCs was associated with tumor size, T stage, TNM stage, vascular invasion, and CEA ↑ M-CTCs: ↑ disease progression: Worse DFS	[122]
	NA	Cyttel method	Negative immune-magnetic selection	≥ 2 CTCs/ 3.2 mL	↑ CTC count in stage III/IV than that of patients in stage I/II CTC count:: metastasis No. of CTCs:: CEA and CA19-9 levels → worse OS & ↑ recurrence rate	[123]
	277	SE-iFISH	CK + /CD45-/DAPI + /CEP8	> 4 CTCs/ 5 mL	CTC were identified as independent prognostic factors for RFS Strongest predictor for the recurrence of stage II CRC patients ↑ CTC levels in patients with poorly differentiated tumors and T4a or T4b stage	[124]
	68	CanPatrol™ CTC enrichment & RNA-ISH	EpCAM, CK8/18/19, Vimentin and Twist	> 5 CTCs/ 5 mL	Total CTCs & M-CTCs post-treatment:: poor PFS & OS	[125]
	356	CytoploRare® Circulating CRC cell kit (Negative enrichment)	FR + CTC & HSP90	11.75 FU	CRC patients with liver metastases showed ↑ expression of FR + CTCs and HSP90 ↑ FR + CTC: ↓ DFS CTCs were risk factors for the progression of metastatic CRC	[94]
	282	NE-imFISH	CEP8 + /DAPI + /CD45 −	≥ 2 CTCs/3.2 mL	CRC patients exhibited significantly ↑ numbers of CTCs Independently associated with poorer DFS and OS Independent predictor of TNM staging, CA-125, and KRAS mutation status	[95]
	79	EpCAM-independent enrichment and CD45- FISH	CEP8 + ,CEP7 + /DAPI + /CD45 −	≥ 2 CTCs/ 3.2 mL	Significantly correlated with clinical response ↑ CTC numbers were associated with a poor treatment response ↑ baseline CTC number was a prognostic factor for unfavorable PFS	[126]
	13 studies (n = 1788 cases)	NA	NA	NA	SV & SP to monitor the recurrence and metastasis value were 67% & 71%, respectively	[127]
	316	RNA-ISH	EpCAM, CK8/18/19, vimentin, and twist	> 3 CTCs/ 5 mL	Total CTC counts were correlated with lymph node involvement More patients with metastasis tested positive for mesenchymal CTCs Platelet/lymphocyte ratio was positively correlated with CTC counts, and lymphocyte/monocyte ratio negatively correlated with CTC counts	[128]
	76	CellRich™ platform system	E-cadherin and vimentin DAPI + /CD45–/CEP8 +	≥ 3 CTCs/ 4 mL	CTC + group: ↓ DFS ↑ CTC count:: Tumor M-stage, tumor location, RAS mutation, ↑ vimentin, & deletion of E-cadherin expression CTC positivity can indicate the efficacy of first-line chemotherapy with oxaliplatin in stage III/IV CRC RAS gene mutation & ↑ vimentin were identified as independent risk factors for a ↑ CTC count	[129]
	101	MiSelect R System	EpCAM + /CK + /DAPI + /CD45-	> 1 CTC/8 mL	Presence of CTCs:: tumor stage Worse DFS & CSS Stratification of patient’s risk status within different stages of disease	[115]
	186	MiSelect R System	EpCAM + /CK + /DAPI + /CD45-	> 5 CTCs/ 8 mL	Presence of CTCs:: Tumor stage, pre-operational CEA, & CA19-9 levels ↑ CTCs:: ↓ OS CTCs + CEA: better OS prediction	[130]
	70	NE-imFISH	NA	0–37	↑ CTCs: ↓ OS Allows patient stratification	[131]
Cervical cancer	107	NE-imFISH	DAPI + / CEP8 + /CD45-	≥ 1 cells/ 3.2 mL	↑ CTCs: ↓ PFS CTC count was an independent negative prognostic factor for survival	[31]
	452	CellSearch™	EpCAM + , CK + , CD45-	7 CTCs/7.5 mL	CTC capture is a potential predictive biomarker to guide treatment selection	[132]
Renal cell carcinoma (RCC)	195	CellSearch™	EpCAM + , DAPI + , & CD45-	> 3 CTCs/7.5 mL	Indicative of shorter OS & PFS	[38]
	163	CanPatrol™ CTC enrichment technique	EpCAM, CK8, CK18, and CK19, Vimentin and Twist	> 9 CTCs/5 mL	Total CTCs, pathology type, and CTC-WBC clusters can be used as prognostic indicators for the MFS of RCC patients Total CTCs can be used as prognostic indicators for the OS of RCC patients ↑ Total CTCs:: worse MFS and OS	[133]
	82	CanPatrol™ CTC enrichment technique	EpCAM, CK8, CK18, and CK19 Vimentin and Twist	Range: 1–104/ 5 mL	M-CTCs:: Platelet count M-CTCs had prognostic value to predict the risk of postoperative metastasis	[134]
Head and Neck squamous cell carcinoma (HNSCC)	83	ISET method	CD45–	2.8 CTCs/mL for OS and 3.8 CTCs/mL for PFS	CTCs counts were an independent prognostic factor for OS & PFS CTM were detected in 27.7% of patients, correlating with worse PFS MRP-7 expression in CTM:: worse OS and PFS CTCs counts were predictive of complete response to treatment	[135]
	95	NE-imFISH	CEP8 + / DAPI + /CD45 −	3 CTCs/ 7.5 mL	↑ CTCs counts: worse prognosis CTCs count:: ↓ PFS, ↓ OS, N stage & clinical stage	[43]
	119	Spiral microfluidic device and IF staining	CK + , DAPI + , CD45-, CSV +	> 1 CTC/ 3 mL	Baseline CTC count:: treatment outcome at 13 weeks post-treatment CSV expression in CTCs:: adverse clinical outcomes	[136]
Glioma	22	SE-FISH	DAPI + , CD45-, and heteroploidy CEP8	> 2 CTCs/ 7.5 mL	↑ Mean CTCs counts in the tumor recurrence group	[84]
	106	FlowSight and FISH	CD45/GFP + /CD56 + , Telomerase positive CTCs	1 CTC/ 4 mL	Presence of postoperative CTCs was a poor prognostic factor Postoperative CTCs were positively correlated with innate immune responses, but negatively correlated with the cytotoxic response	[137]
Biliary tract cancer (BTC)	62	CD-PRIME™ system	EpCAM, Pan-CK, VIM, CK, D45-, DAPI +	V-CTC counts > 50/mL	V-CTC was a significant factor affecting survival	[138]
Lung cancer	51	CellSearch™	EpCAM + , CD45-	≥ 8 CTCs/ 7.5 mL	↑ CTCs (pretreatment, posttreatment or at relapse): worse survival	[139]
	Sixteen cohort studies (n = 1103)	CellSearch™ & others	-	-	↑ Pretreatment CTCs level:: worse OS ↑ Pretreatment CTCs:: ↓ PFS Dynamic monitoring of CTCs level could be a non-invasive & effective tool to predict the disease progression & prognosis in SCLC patients	[112]
	100	Negative enrichment of immune magnetic beads and FISH	CK + /EpCAM + /CD45-/DAPI +	≥ 4 CTCs/ 4 mL	CTC cluster:: disease control rate ↑ CTCs: ↓ OS, ↓ PFS	[140]
	118	Microfluidic Parsortix™ technology	EpCAM, CK19, FAM83A, PTHLH, ERBB3, TWIST1, NANOG, PROM1, MET, BPIFA1, UCHL1 & GRP	NA	EpCAM was the most prevalent transcript, with 53.7% positive samples at primary diagnosis and 25.6% at recurrence EpCAM and CK19, as well as NANOG, PROM1, TERT, CDH5, FAM83A, and PTHLH transcripts, were associated with worse OS However, only the CSC-specific NANOG and PROM1 were related to the outcome both at primary diagnosis and disease progression	[141]
	54	CellSearch™	PD-L1( +) CTCs	> 2 or 5 CTCs/ 7.5 mL	PD-L1( +) CTC:: absence of gene alterations in tumor tissue and with poor prognosis-related biological variables Presence of CTCs and PD-L1( +) CTCs: ↓ OS PD-L1( +) CTCs: poor prognosis in advanced NSCLC patients	[142]
	33	SE-iFISH technology	CEP8, CD44, CD45-, VIM	≥ 12 CTCs/ 6 mL	Extensive-stage SCLC patients: ↑ CTEC counts Detection of CTC-WBC clusters: worse OS & poor response to treatment A high CTC level at B4 was an adverse prognostic factor for SCLC patients	[143]
	391	Anti-CD45/-CD14 immuno-magnetic beads	CD45-/CD14-/FR +	≥ 10.42 FU/ 3 mL	↑ CTC level was a significant VPI risk factor for invasive adenocarcinoma cases	[144]
	59	RosetteSep™ CTC Enrichment Cocktail Containing Anti-CD36	HK2, pan-CK (CK7/8), CD45	≥ 1 CTC/ 5 mL	HK2high/CKneg CTCs display metastasis ↑ HK2high/CKneg CTCs: poor therapy response & ↓ PFS	[145]
	123	EpCAM-CTC-chip	EpCAM + , CK + , CD45-	1 CTC/ mL	Both PFS and cancer-specific survival were predominantly poor Detection of baseline CTCs is a risk factor for recurrence and progression	[146]
	538	Negative enrichment (anti-CD45 & anti-CD14 immunomagnetic beads)	FR + CTC	8.9 FU/ 3 mL	↑ FR + CTC levels in patients with lung cancer than patients with benign disease Increase the efficiency of distinguishing between different histological types of lung cancer and benign space-occupying pulmonary diseases	[107]
	56	CellSearch™ & RossetteSep	EpCAM + , cytokeratins (CKs) 8 + , 18 + , and 19 + , DAPI + , and CD45-	DLA detected 9 times more CTCs than in PB	Sustained presence of CTCs in DLA after treatment: therapy failure & ↓ PFS	[147]
	82	Canpatrol™ CTC technique	EpCAM and CK8/18/19, vimentin and TWIST1	Hybrid CTCs, E-CTCs & M-CTCs per 5 mL blood was 22, 13, & 1	Hybrid E/M-CTCs:: primary tumor size M-CTCs suggested NSCLC progression E-CTCs with a hybrid phenotype: metastasis in therapy-naïve NSCLC patients	[148]
	Meta-analysis (26 studies: n = 1236)	NA	PD-L1 + CTCs	NA	PD-L1 + CTCs: ↓ PFS & OS	[149]
	85	Ficoll-based density gradient centrifugation, Parasortix, OncoQuick, Microfluidic GEN3D6.5 PX separation cassette	panCK, CK19, CD45	0 to > 800	↑ CTC counts & CTC duplets: ↓ OS	[150]
Bladder cancer	Eleven eligible studies (n = 1,062)	–	–	–	CTC-positive patients: poorer survival & ↑ aggressive progression Presence of CTCs: ↑ high risk of mortality	[151]
	196	SE-iFISH	Aneuploid chromosome 8 with DAPI + /CD45 − /CD31 − or diploid chromosome 8 with DAPI + /CD45 − /CD31 − but positively immunofluorescent with 2 tumor TBMs (EpCAM + /VIM + & EpCAM + /VIM −)	≥ 3 CTCs/ 6 mL	CTCs:: clinicopathological characteristics ↑ Triploid CTCs, tetraploid CTCs, and total CECs incipient patients Tumor-biomarker-positive CTCs: worse OS & RFS	[152]
	39	IsoFlux system	CK + /CD45-/DAPI +	≥ 52 CTCs/ 7.5 mL	Independent prognostic biomarker for tumor progression and CSS Allows detection of tumor progression earlier than imaging technique	[153]
	33	IsoFlux™ device	CK + /CD45-/DAPI +	2.3 ± 0.7 to 3.5 ± 1.6/ 7.5 mL	Allowed patient stratification into disease control & progressive disease groups CTC counts were highly correlated with postoperative pathological T stage CTCs possess predictive value for monitoring disease response	[154]
Breast cancer	235	CellSearch™	EpCAM + , CD45-, CK8/18/19 +	≥ 5/ 7.5 mL	CTC count was confirmed to be a robust prognostic marker in the overall population In patients with hEGFR-2 normal tumors, a baseline CTC count ≥ 5 identified subjects who derived benefit from more aggressive treatments	[155]
	3,173	CellSearch™	EpCAM + , CD45-, CK8/18/19 +	≥ 1 CTC/ 7.5 ml	CTC-positive patients: larger tumors, ↑ lymph node involvement, & ↑ histologic tumor grade CTCs was an independent prognostic factor for DFS, distant DFS, breast cancer-specific survival, & OS	[156]
	1,933	CellSearch™	EpCAM + , cytokeratins (CKs) 8 + , 18 + , and 19 + , DAPI + , and CD45-, HER2 status	≥ 1 CTC/ 7.5 ml	CTC status was significantly associated with OS Detection of ≥ 1 CTC with strong HER2 staining was associated with shorter OS	[157]
	164	IMNs	EpCAM + , CK19 + , DAPI + , and CD45-	> 19/ 7.5 mL	CTC positive patients had shorter PFS & OS CTCs detected at baseline or in the late phase of treatment are preferable for prognosis	[158]
	58	CellSearch™	CD45, CK7/8, EpCAM (CD326), CD44, CD24, CD133, N-cadherin (CD325) ApoCTCs: stem and non-stem (CD44 + CD24 − /CD44 − CD24 −)	> 5 CTCs/ 7.5 mL apoCTCs cut-off: 33.55%	The proportion of apoCTCs above the cut-off was an independent prognostic marker of poor metastasis-free survival apoCTC proportion correlates with unfavorable response to neoadjuvant chemotherapy	[159]
	1220	NA	ER + /HER2-, HER2 +	NA	CTC positivity at baseline was associated with shorter OS & DFS CTC positivity predicted early recurrence	[160]
	60	CellSearch™ CXC kit	ER-positive CTCs, EpCAM + /Keratin + /CD45-/DAPI +	Range: 1–207	↑ CTCs during endocrine and chemotherapy:: disease progression CTC-status associated positively with PFS & OS CTC-positivity:: ↓ relapse-free survival	[161]
	52	Pep@MNPs & ICC	CTC-HER2 +	≥ 3 CTCs/ 2 mL	↓ CTCs: ↑ PFS & OS CTC-HER2 status predicted efficacy of anti-HER2 treatment	[100]
	60	CytoploRare® kit	FR + CTC	9.08 FU/ 3 mL	↑ FR + CTC:: disease progression	[101]
	50	CellSearch and TUMORFISH	EpCAM + /CK + /DAPI + /CD45-	CellSearch system: ≥ 1 CTC/ 7.5 mL TUMORFISHER method: ≥ 1 CTC/ 2 mL	CTC enumeration can be a novel assistant biomarker to predict the response of neoadjuvant therapy in patients with HER-2-positive early breast cancer Patients with ≥ 1 CTC before neoadjuvant therapy: ↑ pCR rate ↓ CTC count after neoadjuvant therapy were more likely to achieve pCR	[162]
	137	Pep@MNPs	ER ± , CKmix + /DAPI + /CD45-	> 2 CTCs/ 2 mL	Detection rate for CTCs: 95.71% ↑ CTC ER + : ↑ PFS & OS	[163]
	184	Microcavity array & FACS	panCK + /DAPI + /CD45-	≥ 5 CTCs/ 9.5 mL	After therapy: ↓ CTC count ↑ CTC count:: High-risk patients ↑ CTC count, triple-negative status, and CTC expression of FGFR1:: ↓ PFS & OS Phenotypic plasticity of CTCs:: Poor response to therapy	[164]
	36	Density gradient centrifugation (Histopaque 1077), RT-qPCR	Epithelial panel: EpCAM, CK19, SCGB2A2 Mesenchymal panel: EMP2, SLC6A8, HJURP, MAL2, PPIC, CCNE2	10 Tumor cells/ 5 mL	Positivity rate of mesenchymal panel was higher than epithelial panel CK19 & SCGB2A2:: ↓ OS EMP2, HJURP, MAL2, & CCNE2:: ↓ OS	[165]
	102	CanPatrol™ CTC enrichment technique	EpCAM, CK8/18/19 Vimentin, Twist CD24 + CTCs	94–296 CTCs/5 mL	CD24 + CTCs:: TNM stage, lymph node metastasis, and tumor size Mixed-CTCs: key prognostic indicator for patients with early and intermediate stage	[166]
Pancreatic cancer (PC)	36	CD-PRIME™ platform	EpCAM/pan-CK (CK 8, 18, and 19) and CD45	≥ 1 CTC/ 7.5 mL	CTC positivity was an independent risk factor for early recurrence and for systemic recurrence	[167]
	74	DEP-FFF FACS	EpCAM, CD133, PanCK, CD45-, VIM, pEMT-CTC & SC-CTC	Mean total CTC number: 41.2 ± 62.7	Proportion of pEMT-CTCs:: advanced disease, worse PFS & OS, earlier recurrence after resection	[168]
	40	RosetteSep Human CD45 Depletion Cocktail & Dynal CELLection beads	CK + , CD45-, KLF8 + and VIM + CTCs	Range: 7–71 CTCs/ 7 mL > 50% KLF8 + /vimentin + CTCs	↑ CTCs: ↑ relapse rate The proportion of KLF8 + /vimentin + CTCs to total CTCs was inversely related to TTR > 50% KLF8 + /vimentin + CTCs were identified as independent risk factor	[169]
	104	NA	NA	≥ 1 CTC /mL	↑ CTCs: worse OS mPDAC patients: ↑ CTCs	[170]
	44	CytoploRare Kit	FR + CTCs	14.49 FU/ 3 mL	↑ CTCs:: ↓ DFS, ↑ rate of distant and early recurrence FR + CTCs in resected PC patients predict impaired survival and recurrence patterns after surgery Preoperative CTC levels guide treatment strategies	[171]
	52	CD-PRIME™ system	EpCAM, CK, Plectin-1 and CD45	Median no. of CTCs: 8.5/ 1 mL Range: 0 to 641 cells/ 3 mL	↑ Absolute number of CTCs: ↓ OS ↓ CTCs after chemotherapy: ↑ OS	[108]
	25	CTC isolation Labyrinth (inertial microfluidic technology)	Pan CK, Vimentin, CD45, DAPI +	Range: 0–249 CTCs/mL blood	Number of EMT-CTCs prior to treatment showed a trend towards poorer survival Patients with poor survival tended to have higher mutant KRAS expression in CTCs	[172]
	106	PatrolCTC detection technique and Tricolor RNA-ISH Method	EpCAM, CK8/18/19, vimentin and Twist, and DAPI	≥ 6 CTCs/ 5 mL	Total CTCs, mixed CTCs, & M-CTCs:: shorter PFS Total CTCs and positive M-CTCs:: recurrence & metastasis ↑ CTCs: ↓ CD4, CD4/CD8 ratio, NK cells, IL-2, & IFNγ ↑ CTCs:: weak cellular immunity functions	[173]
Prostate cancer (PCa)	164	CellSearch™	CK + , DAPI + , CD45-	NA	CTC number as a continuous variable is prognostic for survival of patients with CRPC starting first-line chemotherapy ↑ CTC number: ↓ survival time Posttherapy CTC number were most predictive independent of a cutoff value	[174]
	58	Anti-EPCAM immunomagnetic microbead enrichment approach	EpCAM	NA	CTC presence at baseline prior to treatment initiation was independently associated with the risk of nonresponse at 7 months The pooled gene expression from CTCs found AR, DSG2, KLK3, MDK, and PCA3 as genes predictive of nonresponse	[175]
	80	Veridex Cell Search® assay	EpCAM, CK8, CD45 & Ezrin	≥ 5 CTCs/7.5 mL	↑ Expression of Ezrin in CTCs with the metastatic degree	[176]
	3	DLA & AdnaTest	NA	CTCs were only detected in the leukopak, but not in peripheral blood	RNA profiling of DLA CTCs indicated a more aggressive phenotype of CTCs occurred when the patient was experiencing a disease relapse, even when the serum PSA level was relatively low and CTCs in PB were not detectable Provide valuable information for disease monitoring	[177]
	108	ISET® platform	NA	> 1 CTC/ 6 mL	PCa recurrence was significantly associated with the presence of CTC at the preoperative time point CTC presence is an independent risk factor associated with PCa recurrence after prostatectomy	[178]
	74	AdnaTest EMT-2/StemCell kit	EPCAM, EGFR, and ERBB2	NA	CTC expression of AR-V7, AKR1C3, AR, EPCAM, PSMA, MDK, and HPRT1 was significantly associated with OS Baseline CTC biomarkers may be prognosticators for cabazitaxel-treated mCRPC patients	[179]
	104	CanPatrol™ system	EpCAM, CK8/18/19, VIM, twist, CD45 & DAPI, CD133	Median number of baseline CTCs was 4	Can independently predict PFS in metastatic castration-sensitive PCa patient Baseline CTC + CD133 + was a poor independent prognostic factor for metastatic castration-sensitive PCa patients	[180]
	24	CellSearch™, multicolor flow cytometry and imaging cytometry	CD45-EpCAM + panCK + CXCR4 +	> 5 CTCs/ 7.5 mL	Polyclonal CTC population changes after radiotherapy ↑ CTCs:: ↓ PFS ↑ panCK + CXCR4 + CTCs have a prognostic potential in patients with metastatic PCa treated with metastasis-directed radiotherapy	[113]
	87	CellSearch™	Prostein	NA	Prostein + patients: ↓ PSA response rates, PFS, & OS	[181]
	268	NA	PSMA, PSA, AR, AR-V7, EpCAM, KRT 19	NA	PSMA & PSA mRNA were associated with BCR ↑ PSMA & PSA mRNA: ↓ PFS	[182]
Hepatocellular carcinoma (HCC)	179	CanPatrol CTC enrichment technique & RNA-ISH	CD45, EpCAM, CK8/18/19, VIM, Twist	> 5 CTC/ 5 mL	↑ Total CTC & survivin-positive CTC counts: associated with tumor stage & degree of differentiation ↑ Survivin induce HepG2 cell proliferation, ↓ apoptosis, & ↑ invasive ability Survivin-positive CTCs are promising as a predictor of HCC prognosis and metastasis	[183]
	143	CellSearch™	EpCAM based	≥ 1 CTC/ 7.5 mL	↑ Preoperative levels of serum AFP, AFP-L3, & CTCs It has a good clinical value in predicting the short-term efficacy, prognosis and recurrence of HCC patients after MWA	[90]
	153	NE-imFISH	CEP8 + /DAPI + /CD45 −	> 2 CTCs/ 3.5 mL	CTC status was identified as independent predictor and risk factor associated with early recurrence	[184]
	73	Immunoaffinity-based method	EpCAM/ MUC1	≥ 5 CTCs/ 8 mL	↓ CTCs: ↑ survival ↑ Peripheral CTCs:: ↑ tumor aggressive features and poorer survival	[185]
	58	CellSearch™	EpCAM/CK	1.1 ± 1.1/ 7.5 mL	↑ CTCs: ↓ RFS & TTR	[186]
Gastric cancer	116	Human WBC Depletion Kit & FISH Sample Treatment Kit (Cyttel Biosciences)	DAPI + /CD45 − /Chromosome multiploidy (CEP 8 + and/or CEP17 +)	> 3 CTCs/ 3.2 mL	CTC-negative patients: longer mOS Monitoring the change of CTCs is a promising index to evaluate therapeutic effect	[187]
	14 studies (n = 1053 cases)	NA	NA	≥ 2.8 CTCs	CTC-positive patients: ↓ OS & PFS CTC-positive patients: advanced TNM staging, poorer tumor differentiation, & earlier distant metastasis	[188]
	217	CanPatrol™ CTC filtration system 2	CTC-WBC Clusters	CTC-WBC group (n = 29, 13.4%) & CTC group (n = 188, 86.4%)	Positive CTC-WBC cluster patients: ↓ OS CTC-WBC cluster was an independent factor for OS	[189]
	24 studies	NA	NA	31.26% Positivity	Presence of CTCs: worse OS & PFS	[190]
Leptomeningeal metastases (LM)	290	CellSearch™	EpCAM	≥ 61 CSF-CTCs/ 3 mL	↑ CSF-CTC count: ↑ mortality CSF-CTCs quantification predicts survival in newly diagnosed LM, & outperforms neuroimaging CSF-CTC analysis provides quantitative assessment of disease burden in the CNS compartment	[45]
Cholangiocarcinoma (CCA)	38	CellSearch™	EpCAM	Range 1–3 CTCs/ 7.5 mL, median: 1	CTCs were detected in 40% patients & were significantly associated with worse OS CTCs are associated with significantly impaired survival at metastatic stage CTCs detection:: postoperative TNM-status Preoperative CTC detection indicate existing metastases & help to stratify patients	[191]
Multiple myeloma (MM)	374	Next-generation flow cytometry	NA	0.01% CTCs	CTCs were detected in 92% of newly diagnosed MM patients ↑ Logarithmic percentages of CTCs: ↓ PFS CTCs had an independent prognostic value	[192]
Tongue squamous cell carcinoma	50	NanoVelcro system	DAPI + /CK-/CD45 +	≥ 4 CTCs	CTCs levels:: TSCC clinical staging, N staging, & progression status Independent predictive factor for OS & DFS	[53]
Thyroid cancer	394	CanPatrol™ CTC capture technique & tricolor RNA-ISH	EpCAM, CK8/18/19, vimentin and twist	> 6 CTCs/ 5 mL	Positivity rate of CTCs at diagnosis was 95.5% Detection of CTCs and positive M-CTCs: ↓ OS, ↑ early recurrence & ↑ metastasis	[193]
	164	CanPatrol™ capture technique	EpCAM, CK8/18/19, VIM,Twist, & DAPI	> 6 CTCs/ 5 mL	Overall positive rates of CTC at diagnosis were 56.1% ↑ CTCs & M-CTCs: ↓ PFS, ↑ relapse & metastasis rates in PTC and FTC patients	[194]
Esophageal cancer	221	NE-FISH	DAPI + /CD45-/ CEP7/8	≥ 3 CTCs/ 3.2 mL	CTC count:: tumor depth Proportion of CTCs (chromosome 7 triploidy) was linked to distant metastasis & TNM stage ↑ CTCs: ↓ OS	[98]
	96	CellSearch™	EpCAM + , DAPI + , CD45-	1 CTC/ 7.5 mL	↑ CTC count: worse DFS, regional control, distant control, & OS Presence of CTCs in patients during follow-up after tri-modality therapy was associated with poorer DFS and OS	[195]
	88	ClearCell®FX1 System	E-CTCs: DAPI + /CD45-/(pan-CK/EpCAM/MUC1) + M-CTCs: DAPI + /CD45-/vimentin +	> 2–3 CTCs/7.5 mL	↑ CTCs at pre-surgery & pre-treatments Unfavorable pre-surgery CTC status was independent prognostic and predictive for neoadjuvant treatment efficacy CTC clusters are independent prognosticator of tumor relapse CTC clusters and longitudinal CTC monitoring: Potential predictive biomarkers for clinical management	[114]
Neuroblastoma	17	ClearCell® FX microfluidic device	Hoechst + /CD45-	NA	Expression profiles of pro-metastatic genes in CTCs:: presence of bone marrow metastases at diagnosis Persistently ↑ expression of genes in CTCs may serve as novel predictive markers of hematogenous MRD	[196]
Nasopharyngeal carcinoma	93	NA	NA	≥ 8 CTCs/ 3.2 mL	Detection of CTCs:: metastasis & poor OS CTCs demonstrated better SP & SV in predicting prognosis Positive relationship between the detection of CTCs & expression of ALDH1A1 Positive correlation between the CTCs & the expression of CSCs CTCs & CSCs:: metastasis & poor prognosis	[197]
	79	CanPatrol™ CTC enrichment RNA-ISH	EpCAM and CK8/18/19 Vimentin, Twist	> 7 CTCs/5 mL > 5 M-CTCs/5 mL	↑ CTCs & M-CTCs ↑ CTCs & M-CTCs count:: TNM staging, ↓ PFS, & ↓OS	[198]
Osteosarcoma	12	RosetteSep™ CTC Enrichment Cocktail Containing Anti-CD36	HK2high/CD45 − / DAPI +	5 CTCs/ 5 mL	Single CTC test exhibited 100% & 87.5% consistency with therapy response & DFS	[199]
	50	CanPatrol™ CTC enrichment RNA-ISH	EpCAM and CK8/18/19 Vimentin, Twist, IMP3	≥ 1 CTC/5 ml	IMP3 + CTCs:: Enneking Stage of osteosarcoma Ratio of E-type CTCs, M-type CTCs, and H-type CTCs can be used to monitor the therapeutic effect	[200]
Oral squamous cell carcinoma	152	OncoDiscover	EpCAM +	> 3.5 CTCs	Preoperative CTC levels:: adverse clinicopathology factors CTCs indicated nodal metastasis with a linear trend for detecting occult metastasis ↑ CTCs: ↓ median survival & differentiate between early and advanced stages ↑ CTCs: associated with extranodal extension, perineural invasion, and depth of invasion	[99]
Pediatric rhabdomyosarcoma	17	CellSearch™	EpCAM + , CK 8/18/19, DAPI + , & CD45‐	> 1 CTC (median 14.5, range 1–643)	CTCs isolated from blood and bone marrow of RMS patients reflect the molecular alterations found in primary tumor tissues	[201]

↓ decrease, ↑ increase,:: correlate, *AFP* alpha-fetoprotein, *AKR1C3* Aldo–keto reductase 1C3, *ALDH1A1* aldehyde dehydrogenase 1A1, *apoCTCs* apoptotic CTCs, *AR* androgen receptor, *AR-V7* androgen receptor variant v7, *BCR* biochemical recurrence, *BPIFA1* BPI fold containing family A member 1, *BTC* biliary tract cancer, *CA19-9* cancer antigen 19-9, *CCA* cholangiocarcinoma, *CD44* cancer stem cell marker, *CD45* leukocyte common antigen marker, *CDH5* cadherin-5, *CEA* carcinoembryonic antigen, *CEP7/8* chromosome enumeration probe 7/8, *CgA* chromogranin A, *CK* cytokeratin, *CMx* CellMax, *CNS* central nervous system, *CRC* colorectal cancer, *CRPC* castration-resistant prostate cancer, *CSC* cancer stem cell, *CSF* cerebrospinal fluid, *CSS* cancer specific survival, *CTC* circulating tumor cell, *CTEC* circulating tumor-derived endothelial cell, *CTM* circulating tumor microemboli, *DAPI* 4′,6-diamidino-2-phenylindole, *DEP-FFF* dielectrophoresis-field flow fractionation, *DFS* disease free survival, *DLA* diagnostic leukapheresis, *DSG2* desmoglein 2, *E-Cadherin* epithelial cadherin, *EGFR* epidermal growth factor receptor, *EpCAM* epithelial cell adhesion molecule, *ER* estrogen, *ERBB2* erythroblastic oncogene B2, *ERBB3* Erb-b2 receptor tyrosine kinase 3, *FACS* fluorescence-activated cell sorting, *FAM83A* family with sequence similarity 83 member A, *FR* folate receptor, *FTC* follicular thyroid carcinoma, *FU* folate unit, *GFP* green fluorescent protein, *GRP* gastrin-releasing peptide, *HCC* hepatocellular carcinoma, *hEGFR* human epidermal growth factor receptor, *HepG2* human hepatoma cell line, *HER2* human epidermal growth factor receptor 2, *HK2* hexokinase-2, *HNSCC* head and neck squamous cell carcinoma, *HPRT1* hypoxanthine phosphoribosyl transferase 1, *HSP90* heat shock protein 90, *ICC* immunocytochemistry, *IF* immunofluorescence, *IFNy* interferon y, *IL-2* interleukin 2, *IMN* immunomagnetic nanospheres, *IMP3* insulin-like growth factor II m-RNA-binding protein 3, *ISET* isolation by size of tumor cells, *KLF8* Krüppel‑like factor 8, *KLK3* kallikrein-related peptidase 3, *KRAS* Ki-ras2 Kirsten rat sarcoma viral oncogene homolog, *LM* leptomeningeal metastases, *mCRPC* metastatic castration-resistant prostate cancer, *M-CTCS* mesenchymal CTCs, *MDK* midkine growth factor, *MET* c-Met encoding receptor tyrosine kinase, *MFS* Metastasis free survival, *MM* multiple myeloma, *mOS* metastatic overall survival, *mPDAC* metastatic pancreatic ductal adenocarcinoma, *MRD* Minimal residual disease, *MRP-7* multidrug resistance protein 7, *MUC1* mucin 1, *MWA* microwave ablation, *NA* not available, *N-cadherin* neural cadherin, *NE-imFISH* negative enrichment immunofluorescence-in situ hybridization, *NK* natural killer, *NLR* neutrophil to lymphocyte ratio, *NSCLC* non-small cell lung cancer, *OS* overall survival, *PB* peripheral blood, *PC* pancreatic cancer, *PCa* prostate cancer, *PCA3* prostate cancer antigen 3, *pCR* pathologic complete response, *PD-L1* programmed cell death ligand 1, *pEMT* partial epithelial-mesenchymal transition, *PFS* progression free survival, *PLR* platelet lymphocyte ratio, *PROM1* prominin 1, *PSA* prostate-specific antigen, *PSMA* prostate-specific membrane antigen, *PTC* papillary thyroid carcinoma, *PTHLH* parathyroid hormone-like hormone, *RAS* rat sarcoma virus, *RCC* renal cell carcinoma, *RFS* recurrence free survival, *RMS* rhabdomyosarcoma, *RNA-ISH* RNA in situ hybridization, *SC-CTC* stem-cell like CTCs, *SCLC* small cell lung cancer, *SE-iFISH* subtraction enrichment and immunostaining-FISH, *SP* specificity, *SV* sensitivity, *TBM* tumor biomarker, *TERT* telomerase reverse transcriptase, *TNM staging* tumor, nodes, and metastases staging, *TSCC* tongue squamous cell carcinoma, *TTR* time to recurrence, *UCHL1* ubiquitin carboxyl-terminal hydrolase 1, *V-CTCs* vimentin-CTCs, *VIM* Vimentin, *WBC* white blood cell

Further, the functional properties of CTCs such as cluster formation, which indicates their metastatic potential in in vivo conditions, has also been found to be associated with disease progression and reduced OS by in vitro culture of CTCs isolated from lung [140, 143], gastric [189], and esophageal [114] cancer patients. Also, a number of studies have suggested the prognostic significance of the molecular phenotypes of CTCs. Prognostic value of EpCAM-positive CTCs has been reported in precancerous and cancerous lesions of breast, prostate, bladder, and kidney cancer [202, 203]. Additionally, the EpCAM-positive CTCs can predict both early distant metastasis as well as worse OS [204–206]. The presence of CTCs expressing mesenchymal or stemness-related markers, have been found to be associated with inferior survival in CRC [120, 122], RCC [134], lung cancer [148], pancreatic cancer [173], thyroid cancer [193, 194], and NPC [198]. Interestingly, in some studies technological variations in CTC detection did not impact prognostic interpretations [207]. For instance, CTCs as measured by CellSearch represent an independent prognostic factor determining OS and PFS [208]. However, studies evaluating prognostic value of CTCs using other CTC detection systems like CanPatrol [209] or CTC-chip [146] obtained similar results. These studies show a strong positive predictive value (PPV) of CTCs. It is also important to highlight that most of these studies emphasized the PPV of CTCs, however, the study design often lacked assessment of negative predictive value (NPV) of CTCs, which are consistently lower than PPVs in some sporadic studies [88, 210–212]. In such scenarios, most of these tests can be used as confirmatory tests only and not as a standalone marker to rule out metastases. Further, there is contrasting report on CRC, wherein the presence of CTCs showed no significant correlation with clinicopathological features and was not predictive of DFS or OS [117]. This was attributed to the scarcity of CTCs in stage III CRC patients, which could be possibly attributed to lower SV of the technique. Nevertheless, the enumeration and characterization of CTCs provide real-time, minimally-invasive monitoring of cancer, and guide treatment decisions. It also acknowledges the need for improvement in SV of CTC-detection technologies and large-scale validation studies for clinical translation.

### CTCs as a tool for therapeutic monitoring

With increasing understanding of CTCs, CTC numbers have proved to be effective biomarker for monitoring therapeutic response in clinical setup [213–215]. Since, detection of CTCs being a minimally-invasive method, it minimizes the necessity for frequent exposure to radioactive imaging or sampling the tumor site. A correlation between CTC count before and after the intervention revealed the efficacy of the treatment plans. Studies have reported that increased CTC counts indicate poor therapeutic response, while decreased counts indicate a positive therapeutic response as summarized in Table 5.

**Table 5:** Circulating tumor cells as a biomarker for therapeutic monitoring for different types of cancers

Cancer type	Sample size (n)	Volume of blood (mL)	CTC enrichment technique	Nature of marker used	Therapeutic intervention	CTC Range Pre-treatment	CTC Range Post-Treatment	Refs.
Breast Cancer	235	7.5 mL	CellSearch™	EpCAM CD45	Endocrine treatment Monochemotherapy Combination Chemotherapy Chemotherapy + anti-HER2 drugs Chemotherapy + bevacizumab	CTC < 5 (n = 141) CTC ≥ 5(n = 94)	CTC < 5 (n = 33), CTC ≥ 5 (n = 14) CTC < 5 (n = 23), CTC ≥ 5(n = 22) CTC < 5(n = 40), CTC ≥ 5(n = 24) CTC < 5(n = 26), CTC ≥ 5(n = 14) CTC < 5(n = 19), CTC ≥ 5(n = 20)	[155]
	160	7.5 mL	CellSearch™	INHBA	Surgical Trastuzumab	79.64 ± 21.06 81.29 ± 23.94	58.33 ± 15.92 (1 month after chemotherapy) 18.76 ± 9.73 (3 months after chemotherapy) 69.64 ± 18.61 (1 month after chemotherapy) 37.91 ± 14.88 (3 months after chemotherapy)	[214]
Small Cell Lung Cancer	51	7.5 mL	CellSearch™	EpCAM CD45	Chemotherapy Radiotherapy	CTCs: Median:4, Range: 0–5648	CTCs: Median:0, Range: 0–253	[139]
	42	7.5 mL	CellSearch™	EpCAM DAPI CK CD45	At chemotherapy At 2 months follow-up	CTCs: Median:11, Range: 0–4434	CTCs: Median:0, Range: 0–452 CTCs: Median:0, Range: 0–280	[216]
Neuroblastoma	40	4–8 mL	Amnis Image Stream Imaging flow Cytometer (ISx)	GD2 CD45 NCAM DAPI	Nutlin-3	1–264 CTC/mL	1–39 CTC/mL Despite the decrease in CTC count/mL, relapse occurred	[33]
Prostate cancer	65	7.5 mL	CellSearch™	EpCAM	After neoadjuvant androgen deprivation therapy and before radiotherapy After Radiotherapy 6–12 months after radiotherapy	*% positivity: 7.5%, CTC range: 1–6* Cutoff: ≥ 1 cells/7.5 ml	*% positivity: 12.9%, CTC range: 1–1* *% positivity: 18.6%, CTC range: 1–136* *% positivity: 7.6%, CTC range: 1*	[217]
Colorectal Cancer	100	7 mL	Flowcytometry	CK-19 CD45	Surgery Chemotherapy Radiotherapy	8.89 ± 10.8 CTCs (Mean ± SD)	6.26 + 8.5 CTCs (Mean ± SD)	[111]
Glioblastoma	20	NA	Spiral microfluidic technology, ICC	CSV, CD45 GFAP	Surgery	0–5 CTCs (Range)	0–5 CTCs (Range)	[41]
Head and Neck Squamous Cell Cancer	21	10 mL	RosetteSep™	CK N-Cadherin CD45 DAPI	DCF mDCF	0–0.880 CTCs/mL (Median) 0.125–3.335 CTC/mL	0–1.505 CTCs/mL (Median) NA	[46]
Penile Squamous cell carcinoma	14	NA	IsoFlux CTC enrichment with CELLection Dynabeads	EpCAM IgG	Surgery	3.4 ± 2.1 (Mean ± SD)	1.2 ± 1.1 (Mean ± SD)	[47]

*CD* cluster of differentiation, *CK *cytokeratin, *CSV* cell surface vimentin, *CTC* circulating tumor cell, *DAPI* 4′,6-diamidino-2-phenylindole, *DCF *Docetaxel, Cisplatine, 5-Fluorouracil, *EpCAM* epithelial cell adhesion molecule, *GD*_*2*_ disialoganglioside, *GFAP* Glial fibrillary acidic protein, *INHBA* Inhibin, beta A, *mDCF* DCF modified (dose adapted), *NCAM* neural cell adhesion molecule

In metastatic breast cancer, a sample of 235 patients was analyzed using 7.5 mL of blood with the CellSearch™ technique, focusing on EpCAM and CD45 markers. Different treatments were administered, and pre-treatment CTC levels were categorized as either below or above 5, with varying post-treatment CTC ranges observed across different therapeutic interventions [155]. Another report of breast cancer comprising of 160 patients using the CellSearch technique demonstrated a decrease in CTCs following surgical and trastuzumab interventions [214]. For SCLC, 51 patients were evaluated with 7.5 mL of blood, showing a significant reduction in CTC range post-chemotherapy and radiotherapy [139]. NPC in 60 patients utilized a 5 mL blood sample enriched via the CanPatrol technique, with a decrease in CTC levels correlated with treatment [29]. In neuroblastoma, 40 patients’ CTC levels were assessed using the Amnis Image Stream Imaging Flow Cytometer, showing a notable drop post-treatment with Nutlin-3 [33]. Prostate cancer in 65 patients showed fluctuating CTC levels before and after various treatments [217], while CRC in 100 patients revealed a general decrease post-surgery, chemotherapy, and radiotherapy [111]. Glioblastoma [41], HNSCC [46], and penile squamous cell carcinoma [47] studies indicated variable CTC levels before and after respective treatments, with most showing a decrease post-intervention. Overall, the findings illustrated the potential of CTCs as biomarkers for monitoring therapeutic efficacy across diverse cancer types. Evidences suggest a strong correlation between declining CTC counts and favorable therapeutic outcomes, while persistent or elevated counts are indicative of poor response. Studies using various detection platforms including CellSearch, flowcytometry, spiral microfluidic technology, RosetteSep™, and IsoFlux have consistently demonstrated treatment-associated fluctuations in CTC counts across cancers such as breast, lung, prostate, colorectal, glioblastoma, HNSCC, neuroblastoma, and penile squamous cell carcinoma. Despite promising trends, a definitive correlation between CTC counts before and after treatment remains largely unexplored in most literature, posing a significant gap in understanding. This gap underscores the need for longitudinal, large-scale studies to validate CTCs as reliable markers of treatment response and to refine their clinical application.

## Opportunities and challenges in CTC-directed clinical utility

### Opportunities

To sum up wide spectrum of opportunities offered by the CTCs, some striking ones are highlighted below.

*Diagnostics*: CTCs primarily aid in early cancer diagnosis and assessing tumor progression.

*Prognostication*: Irrespective of the technologies used, elevated CTC counts have been consistently associated with poor prognosis across several cancers, supporting their role as independent prognostic markers. CTC count and positivity enable effective patient stratification, provide valuable information about treatment response rates, guides treatment selection, and help assess the risk of disease recurrence.

*Therapeutic monitoring*: Using this minimally-invasive technique, serial enumeration and molecular profiling of CTCs during therapy in various cancers is made feasible. The CTC count offer insights into tumor dynamics, treatment efficacy, and emergence of resistance supporting treatment personalization and timely modification of therapy.

*Access to deep seated malignant tumors***:** CTCs enable early detection of cancers, particularly those originating from deep-seated tumors, effectively bridging the gap between tumor progression and late diagnosis, especially in rapidly advancing cancers[218].

*Live tumor cell detection*: CTCs provide minimally-invasive access to viable, intact tumor cells, irrespective of the tumor site of origin, offering real-time molecular and cellular profiling without requiring invasive tumor biopsies.

*Transcriptomic profiling for assessing CTC heterogeneity and detection of metastatically-competent CTCs*: Live CTC capture and recovery followed by their dissection at genomic, transcriptomic, proteomic, and epigenomic levels, can reveal biologically aggressive CTC subsets with high metastatic potential.

*CTC-based* in vitro* cultures and* in vivo *tumor models*: Isolation of live CTCs further opens window of opportunity leading to establishment of primary CTC cultures and CDX models that permit functional validation of drug response, understanding drug resistance mechanisms, and help predicting tumor evolution under therapeutic pressure.

*Selection-free CTC enrichment*: Advancements in label-free or hybrid technologies enable the capture of CTCs without selection bias towards specific markers like EpCAM, thus preserving sub-populations (e.g., mesenchymal or stem-like CTCs) critical for understanding metastasis. Label-free technologies such as Isolation by SizE of Tumor (ISET), density gradient centrifugation, RosetteSep, ClearCell® FX microfluidic device, and Parasortix, have emerged as an alternative to capture a wide spectrum of heterogenous CTCs. Hence, hybrid technologies such as CanPatrol™ have reported to exhibit enhanced CTC enrichment.

### Challenges

*Necessity of large-scale prospective validation studies*: The existing literature on the clinical potential of CTCs remains limited, as most studies are retrospective in nature or based on small patient cohorts, leading to increased risk of bias and inconsistent or incomplete data. To establish the true clinical relevance of CTCs and enable their integration into routine practice, well-designed prospective, longitudinal, and multi-center trials are crucial [51, 181]. Such studies, especially those with extended follow-up periods [117], will offer greater control over data quality and allow for more robust correlations between CTC counts and clinical disease manifestations.

*Cost-effectiveness of CTC-based diagnostics compared to alternative biomarkers*: In high-resource economies, such as United States, CTC-guided selection of first-line therapy in HR + /HER2- metastatic breast cancer has been shown to be cost-effective from the US payer’s perspective [219]. However, in developing and underdeveloped countries, the economic feasibility of CTC-based diagnostics is significantly challenged by the high cost of detection platforms, limited healthcare infrastructure, and delayed clinical intervention. For instance, CellSearch™ test, only FDA-approved CTC-detection technology, costs around $350 to $900 per test [220, 221]. In the Indian clinical context, available CTC detection assays such as ISET [222], OncoPro Colorectal Cancer Screen CTC Test [223], and OncoPro PD-L1 CTC test [224] are priced at $234, $410, and $684 respectively. Hence, despite the promising clinical value of CTCs, relatively higher costs of these CTC tests hinder their routine clinical adoption in low-resource settings. In contrast, the conventional serum biomarkers such as CEA, CA-125, or prostate-specific antigen (PSA) typically cost between $3 to $30, depending upon the geopgraphical region. Similarly, imaging modalities such as PET-CT scans range from $110 to $300, with subsidized rate equivalent to as low as $2 at government hospitals. Although these traditional methods are more accessible and cost-effective, they often lack the specificity and real-time molecular resolution offered by CTCs. To bridge this gap between economic barrier and clinical translation, the field of CTC requires potential technological innovations that reduce the cost of reagents and instruments involved. Label-free microfluidic technologies such as CTC-cluster chip, Parasortix, RUBY-chip, and ieSCI chip that depend on biophysical properties of CTCs rather than expensive antibodies, have emerged as cost-effective alternatives. In conjugation, these platforms provide semi-automated workflows that improves reproducibility and scalability. Furthermore, upcoming technologies such as paper-based microfluidic systems offer minimal sample requirement, ease of use, and portability that can be better adapted for point-of-care testing [225]. However, the SV of these platforms remain a key question. These low-cost platforms could reduce per-test costs, expand CTC utility in low-resource settings, and eventually accelerate the integration of CTCs into routine oncology practice globally.

*Influence of pre-analytical variables on CTC detection and enumeration*: Variables such as blood collection tubes, anticoagulant type, amount of blood drawn, processing time, and storage conditions can significantly affect CTC yield and integrity. Diurnal variations, treatment cycles, and physiological factors can influence CTC levels [226]. However, related investigations are still lacking that highlight the need for standardization of such pre-analytical variables.

*Rare population*: CTCs are extremely scarce (as few as 1–10 CTCs per 10⁹ blood cells or 1–100 CTCs per mL of blood), making their reliable detection and analysis technically challenging and often requiring highly sensitive platforms. This continues to be a major bottleneck in achieving reliable proficiency in CTC quantification and analysis.

*CTC heterogeneity*: CTCs exhibit significant phenotypic and genotypic diversity due to processes like EMT that contribute to cellular plasticity making it difficult to define a single biomarker or enrichment strategy for all subtypes. The metastatic potential of CTC is still unclear. This demands functional validation of CTCs to capture metastasis-competent sub-population of CTCs for tapping the aggressive biologically relevant CTCs responsible for metastasis.

*Technological variations and lack of standardization/harmonization across different CTC detection platforms*: The diverse range of CTC detection and isolation platforms such as CellSearch, microfluidics-based systems, and size-based filtration exhibit varying capture efficiencies and sensitivities across different malignancies [22]. This variability poses significant challenges in standardizing methodologies and harmonization of the end results, hindering cross-study comparisons, meta-analyses, and ultimately, the clinical translation of CTC-based approaches. To overcome this, establishment of a more structured framework is required towards standardization. The standardization procedure should involve prioritizing pre-analytical variables such as blood collection tubes with type of anticoagulant, volume of sample required, time of sample processing, and sample storage conditions. These parameters impact CTC yield and viability that needs harmonization for better reproducibility. This should be followed by standardization of different CTC isolation and enrichment efficiency metrics including tumor cell recovery efficiencies, purity, and throughput using preferably (but not limited to) established cancerous cell lines spiked-in experiments to mimic patient samples in a controlled manner. Lastly, the reporting of generated data and its interpretation including CTC defining characteristics such as marker expression or CTC enumeration thresholds require malignancy-specific standardization. Additionally, establishment of international consortia similar to the International Society of Liquid Biopsy (ISLB), Liquid Biopsy Consortia (LBC), International Liquid Biopsy Standardization Alliance (ILSA), the BloodPAC Consortium, and the European Liquid Biopsy Society (ELBS) might facilitate the procedure of establishing CTC-specific clinical benchmarks by exchange of information globally. Based on the current gaps in the field of CTC, analytical validation should be prioritized first rather than clinical validation. Establishment of robust and reproducible technologies for CTC detection, isolation, and characterization is a prerequisite for downstream clinical validation and for valid comparison of outputs over a period of time for patient monitoring.

*Undersampling of CTC population*: Current CTC detection and isolation technologies are primarily based on EpCAM. Despite being highly specific, they have variable sensitivities. Among the different technologies, EpCAM-based immunocapture technology, i.e., CellSearch, stands the only FDA-approved technology, whereas other CTC detection and isolation techniques still await regulatory approvals and are still far from direct clinical application. Selection-bias induced by such marker-based capture of CTCs have often resulted in undersampling of these cells. Certain reports have shown that actual CTC count is 30–100 folds higher than estimated by CellSearch alone [227, 228]. However, both label-dependent and label-independent platforms introduce parametric-bias (marker-based selection or physiological property) that prevents isolation of entire population of CTCs [22]. This highlights the need for sensitive and robust technology to capture actual burden of CTCs.

*Lack of diagnostic thresholds for clinical implementation*: Despite promising correlations, universally-accepted CTC thresholds for diagnosis and treatment decision-making are still lacking. A randomized controlled trial in stage II colon cancer established ctDNA thresholds as decision-making yardstick for administering adjuvant chemotherapy and showed patients with no detectable ctDNA post-treatment had 97% chances of RFS up to 5 years (ACTRN12615000381583) [229, 230]. This highlights how circulating biomarkers have successfully navigated the transition to clinical implementation and provides a compelling parallel to how CTCs count and characterization from exploratory phase can translate into clinically meaningful biomarkers. Further, there are no tailor-made controls, particularly negative controls. To address this, as emphasized earlier, routine in vitro spike-in controls [231] should be included across different CTC detection platforms to validate the SV of testing following which robust clinical correlation studies may help establish stage-specific and platform-wise thresholds for clinical application. In addition, as there is accumulation of larger platform-wise datasets, meta-analytical approaches combining data from multiple studies will aid in establishment of cancer-specific CTC-cutoff values. For instance, CellSearch platform in metastatic breast cancer that have validated ≥ 5 CTCs per 7.5 mL of blood as a clinically meaningful benchmark threshold for prognostic stratification [232, 233].

*Potential limitation of statistical approaches being employed for CTC-based analyses*: Statistical analyses involving CTCs must account for several critical variables, including the adequacy of CTC frequency to justify treating the sample size as infinite, the impact of cell loss during processing on detection probability, the representativeness of aliquots for the entire blood sample, and the consistency of results across assays using varying blood volumes [234]. Further small sample sizes and multiple-hypothesis testing amplify data variability. Current statistical models may not adequately account for CTC variability, limiting predictive accuracy. Additionally, rather than consolidating or quantitatively advancing the field, many meta-analyses on CTCs are hindered by methodological inconsistencies, limited sample sizes, and selective reporting, potentially leading to overestimated clinical relevance and utility.

*Lack of trained manpower*: The detection and analysis of CTCs involve complex procedures that necessitate specialized training and expertise. Advanced techniques such as immunofluorescence microscopy, flowcytometry, sophisticated CTC-detection and isolation strategies, and single-cell omic analyses are integral to CTC measurements, requiring proficiency in technical execution and interpretation of complex data. Hence, CTC-related research demands a highly skilled workforce with strong expertise in molecular biology techniques, hands-on experience with advanced CTC-detection and isolation technologies, and expertise in data analysis and interpretation. The scarcity of such multidisciplinary training poses a significant barrier to progress in this field.

## Conclusion and future prospects

Although CTCs provide crucial insights into cancer metastasis, rare and heterogeneous nature of these cells poses a major challenge in their isolation, detection, and characterization. Improvements in their isolation and detection strategies with time, including both label-dependent as well as label-independent, have enhanced the clinical utility of CTCs. Still dynamic nature of CTCs stemming from EMT prevents their effective utilization. Current evidence indicates that CellSearch™, an EpCAM-based immunomagnetic positive selection system, remains the most widely utilized approach for the detection and isolation of CTCs. This prevalence can be largely attributed to its status as the only FDA-approved technology in this domain, as well as its extensive validation across various cancer types. Being a closed system, it poses a significant constraint in harnessing the potential of this valuable biomarker in low-resource settings. Growing understanding of CTC heterogeneity, particularly the presence of mesenchymal and stem-like subpopulations, has shifted the dynamics towards alternative strategies. These include both marker-dependent technologies (targeting markers such as CKs, CD44, FR, vimentin, Twist, N-cadherin, and CD133) and label-free methods (such as ISET, Parsortix™, spiral microfluidic devices, ficoll-based density gradient centrifugation, and the ClearCell® FX system). Recently, Parasortix™ has received FDA approval for isolation of CTCs in metastatic breast cancer. While these emerging platforms offer promising avenues to capture a broader spectrum of CTC phenotypes, they remain largely in the exploratory phase and require further standardization and large-scale clinical validation to be incorporated into routine clinical practice. This highlights the need of simpler and more reproducible assays where heterogeneity of cells could be maintained. To fill in the translational gap in clinical utility of CTCs, firstly, the standardization and validation of marker-independent isolation strategies should be accomplished to harness their advantage for capturing the full heterogeneity of CTC populations, including mesenchymal and stem-like subsets often missed by marker-dependent methods. Second, the functional assays, such as spheroid formation or in vivo tumorigenicity models, should be incorporated to identify rare metastasis-initiating CTCs with true biological relevance. Third, distinct CTC subpopulations hence identified and characterized should be correlated with therapeutic response profiles to refine treatment stratification. Also, integrating multi-omics approaches into traditional strategies will aid in dissecting the molecular profile of CTCs for identification of aggressive population of CTCs and patient stratification. In addition, culturing of CTCs in vitro and development of CDX in vivo model can further help in examining their seeding capacity and dose-dependent response of therapeutic strategies for targeting these tumor cells in circulation. Hence, CTCs hold a great potential for enhancing our understanding on carcinogenic progression and distant metastases while improving the patient care. With ongoing research and technological innovations, CTCs are set to become an integral component of precision oncology, offering hope for more effective and personalized cancer treatments.

## Significance

A comprehensive overview of CTC parameters in vitro and in vivo, along with their current clinical utility will help in understanding the present-day extent to which the clinical potential of CTCs is getting tapped in personalized medicine and what gaps remain for their broader clinical application. This understanding is crucial for developing standardized, clinically-viable strategies that can overcome current limitations and translate CTC-based technologies into routine oncology practice

## Supplementary Information

Supplementary file 1.

## Data Availability

No datasets were generated or analysed during the current study.
