# Supplementary material for: Redefining cancer care: harnessing circulating tumor cells’ potential for improved diagnosis and prognosis
Source: Cancer Cell Int. 2025 Jul 17;25:267. doi: 10.1186/s12935-025-03883-y (PMC12273008; doi:10.1186/s12935-025-03883-y)
Supplement: Supplementary file 1 [file 12935_2025_3883_MOESM1_ESM.docx]

**Supplementary table ST1: Metadata of clinical studies compiled for representative plot of cancer-specific CTC count as reported (Figure 1).**

| **Cancer** | **CTC count (Median/Mean/Range)** | **CTC isolation platform** | **Ref.** |
| --- | --- | --- | --- |
| **Anal cancer** | 60–876 cells/mL | Amnis® ImageStream®X Mk II Imaging Flow Cytometer | [1] |
| **Bladder cancer** | 0-29 CTCs/5mL | CanPatrol^TM^ system | [2] |
| **Breast cancer** | 25 to 10,000 cells/mL | ZnFe_2_O_4_ nanoparticles | [3] |
| **Cervical cancer** | 0 to 27 cells in 3.2 mL blood | NE-imFISH | [4] |
| **Cholangiocarcinoma** | 0-25 CTCs/7.5mL | CellSearch^TM^ | [5] |
| **Colon cancer** | 0-94 cells/8 mL | ISETⓇ (Isolation by SizE of Tumor cells) | [6] |
| **Colorectal cancer** | 0-9 CTCs/7.5mL | NE-imFISH | [7] |
| **Gallbladder cancer** | 0-4 CTCs/7.5mL | CellSearch^TM^ | [5] |
| **Gastric cancer** | 7.41-1147 cells/10mL | FACS | [8] |
| **Glioblastoma** | 1-24 cells/mL | Cytospin™ Spiral chip | [9] |
| **Head and neck squamous cell carcinoma** | 0.38 ± 0.72 and 3.77 ± 2.82 (CTCs/7.5 ml) | NE-imFISH | [10] |
| **Leptomeningeal metastases** | 0-200 CTCs/3mL | CellSearch^TM^ | [11] |
| **Malignant Pleural Mesothelioma** | 0-11 CTCs/mL | Universal CTC-chip | [12] |
| **Melanoma** | 0-2.298 ± 0.159/mL | Cytophone platform | [13] |
| **Multiple myeloma** | 63-499 CTCs/mL | Epic Platform | [14] |
| **Nasopharyngeal carcinoma** | 3-52 CTCs/5mL | CanPatrol^TM^ system | [15] |
| **Neuroblastoma** | 1-264/mL | Imagestream Imaging flow cytometer (ISx) | [16] |
| **Non-Hodgkin lymphoma** | 0 - 17,813 cells/ml | Real time PCR | [17] |
| **Non-small cell lung cancer** | 0-31 cells/7.5mL | Not disclosed | [18] |
| **Oesophageal cancer** | 0–150 CTCs/7.5 mL | Combined MACS Enrichment Method | [19] |
| **Oropharyngeal cancer** | 0.07 - 3.34 CTC/mL | RosetteSep^TM^ technique | [20] |
| **Ovarian cancer** | 0-19 cells/4mL | V-BioChip (CytoAurora Inc., HsinChu, Taiwan) | [21] |
| **Pancreatic cancer** | 1-7.75 cells/7.5mL | High-density microporous chip (SMART BIOPSYTM Cell Isolator; Cytogen Inc., Seoul, Korea) | [22] |
| **Penile squamous cell carcinoma** | 2-6.3 CTCs/7.5mL | MACS | [23] |
| **Prostate cancer** | 5 to 854 cells/7.5 mL | CellSearch^TM^ | [24] |
| **Renal cell carcinoma** | 1-263 CTCs/7.5mL | CellSearch^TM^ | [25] |
| **Small cell lung cancer** | 5.8-83.3 cells/mL | EasySep™ Direct Human CTC Enrichment Kit/ FACS | [26] |
| **Testicular cancer** | 1-14 CTCs/7.5mL | CellSearch^TM^ | [27] |
| **Thyroid cancer** | 0-72 cells/7.5mL | NE-imFISH | [28] |

**REFERENCES**

1. Carter TJ, Jeyaneethi J, Kumar J, Karteris E, Glynne-Jones R, Hall M: **Identification of Cancer-Associated Circulating Cells in Anal Cancer Patients**. *Cancers* 2020, **12**(8).

2. Zhang R, Xia J, Wang Y, Cao M, Jin D, Xue W, Huang Y, Chen H: **Co-Expression of Stem Cell and Epithelial Mesenchymal Transition Markers in Circulating Tumor Cells of Bladder Cancer Patients**. *OncoTargets and therapy* 2020, **13**:10739-10748.

3. Vajhadin F, Mazloum-Ardakani M, Hemati M, Moshtaghioun SM: **Facile preparation of a cost-effective platform based on ZnFe(2)O(4) nanomaterials for electrochemical cell detection**. *Scientific reports* 2023, **13**(1):4962.

4. Du K, Huang Q, Bu J, Zhou J, Huang Z, Li J: **Circulating Tumor Cells Counting Act as a Potential Prognostic Factor in Cervical Cancer**. *Technology in cancer research & treatment* 2020, **19**:1533033820957005.

5. Al Ustwani O, Iancu D, Yacoub R, Iyer R: **Detection of circulating tumor cells in cancers of biliary origin**. *Journal of gastrointestinal oncology* 2012, **3**(2):97-104.

6. Abdallah EA, Souza ESV, Braun AC, Gasparini VA, Kupper BEC, Tariki MS, Tarazona JGR, Takahashi RM, Aguiar Junior S, Chinen LTD: **A higher platelet-to-lymphocyte ratio is prevalent in the presence of circulating tumor microemboli and is a potential prognostic factor for non-metastatic colon cancer**. *Translational oncology* 2021, **14**(1):100932.

7. Yu JH, Wang D, Jin L, Wang J, Zhao XM, Wu GC, Yao HW, Yang YC, Zhang ZT: **Utility of circulating tumor cells in stage II colorectal cancer patients undergoing curative resection**. *Translational cancer research* 2020, **9**(3):1487-1494.

8. Miki Y, Yashiro M, Kuroda K, Okuno T, Togano S, Masuda G, Kasashima H, Ohira M: **Circulating CEA-positive and EpCAM-negative tumor cells might be a predictive biomarker for recurrence in patients with gastric cancer**. *Cancer medicine* 2021, **10**(2):521-528.

9. Muller Bark J, Kulasinghe A, Hartel G, Leo P, Warkiani ME, Jeffree RL, Chua B, Day BW, Punyadeera C: **Isolation of Circulating Tumour Cells in Patients With Glioblastoma Using Spiral Microfluidic Technology - A Pilot Study**. *Frontiers in oncology* 2021, **11**:681130.

10. Zhou S, Wang L, Zhang W, Liu F, Zhang Y, Jiang B, Wang J, Yuan H: **Circulating Tumor Cells Correlate With Prognosis in Head and Neck Squamous Cell Carcinoma**. *Technology in cancer research & treatment* 2021, **20**:1533033821990037.

11. Diaz M, Singh P, Kotchetkov IS, Skakodub A, Meng A, Tamer C, Young RJ, Reiner AS, Panageas KS, Ramanathan LV *et al*: **Quantitative assessment of circulating tumor cells in cerebrospinal fluid as a clinical tool to predict survival in leptomeningeal metastases**. *Journal of neuro-oncology* 2022, **157**(1):81-90.

12. Kuwata T, Yoneda K, Mori M, Kanayama M, Kuroda K, Kaneko MK, Kato Y, Tanaka F: **Detection of Circulating Tumor Cells (CTCs) in Malignant Pleural Mesothelioma (MPM) with the "Universal" CTC-Chip and An Anti-Podoplanin Antibody NZ-1.2**. *Cells* 2020, **9**(4).

13. Galanzha EI, Menyaev YA, Yadem AC, Sarimollaoglu M, Juratli MA, Nedosekin DA, Foster SR, Jamshidi-Parsian A, Siegel ER, Makhoul I *et al*: **In vivo liquid biopsy using Cytophone platform for photoacoustic detection of circulating tumor cells in patients with melanoma**. *Science translational medicine* 2019, **11**(496).

14. Zhang L, Beasley S, Prigozhina NL, Higgins R, Ikeda S, Lee FY, Marrinucci D, Jia S: **Detection and Characterization of Circulating Tumour Cells in Multiple Myeloma**. *Journal of circulating biomarkers* 2016, **5**:10.

15. Wen Z, Li Z, Yong P, Liang D, Xie D, Chen H, Yang Y, Wu S, Li C, Cheng Z: **Detection and clinical significance of circulating tumor cells in patients with nasopharyngeal carcinoma**. *Oncology letters* 2019, **18**(3):2537-2547.

16. Merugu S, Chen L, Gavens E, Gabra H, Brougham M, Makin G, Ng A, Murphy D, Gabriel AS, Robinson ML *et al*: **Detection of Circulating and Disseminated Neuroblastoma Cells Using the ImageStream Flow Cytometer for Use as Predictive and Pharmacodynamic Biomarkers**. *Clinical cancer research : an official journal of the American Association for Cancer Research* 2020, **26**(1):122-134.

17. Debled M, Hostein I, Astier-Gin T, Eghbali H, Bonichon F, Soubeyran I, Richaud P, Hoerni B, Soubeyran P: **Competitive polymerase chain reaction to quantify tumor cells in peripheral blood of patients with T(14;18)-bearing follicular non-Hodgkin's lymphoma: an exploratory study in 8 patients**. *International journal of cancer* 1999, **84**(6):558-561.

18. Manjunath Y, Suvilesh KN, Mitchem JB, Avella Patino DM, Kimchi ET, Staveley-O'Carroll KF, Pantel K, Yi H, Li G, Harris PK *et al*: **Circulating Tumor-Macrophage Fusion Cells and Circulating Tumor Cells Complement Non-Small-Cell Lung Cancer Screening in Patients With Suspicious Lung-RADS 4 Nodules**. *JCO precision oncology* 2022, **6**:e2100378.

19. Woestemeier A, Harms-Effenberger K, Karstens KF, Konczalla L, Ghadban T, Uzunoglu FG, Izbicki JR, Bockhorn M, Pantel K, Reeh M: **Clinical Relevance of Circulating Tumor Cells in Esophageal Cancer Detected by a Combined MACS Enrichment Method**. *Cancers* 2020, **12**(3).

20. Gauthier A, Philouze P, Lauret A, Alphonse G, Malesys C, Ardail D, Payen L, Ceruse P, Wozny AS, Rodriguez-Lafrasse C: **Circulating Tumor Cell Detection during Neoadjuvant Chemotherapy to Predict Early Response in Locally Advanced Oropharyngeal Cancers: A Prospective Pilot Study**. *Journal of personalized medicine* 2022, **12**(3).

21. Jou HJ, Chou LY, Chang WC, Ho HC, Zhang WT, Ling PY, Tsai KH, Chen SH, Chen TH, Lo PH *et al*: **An Automatic Platform Based on Nanostructured Microfluidic Chip for Isolating and Identification of Circulating Tumor Cells**. *Micromachines* 2021, **12**(5).

22. Choi YH, Hong TH, Yoon SB, Lee IS, Lee MA, Choi HJ, Choi MH, Jung ES: **Prognostic Implications of Portal Venous Circulating Tumor Cells in Resectable Pancreatic Cancer**. *Biomedicines* 2022, **10**(6).

23. Kao CC, Xu T, Yang YN, Tsai YT, Liu SY, Wu ST, Meng E, Tsao CW, Chen CL, Sun GH *et al*: **Detection of circulating tumor cells as therapeutic markers in patients with penile squamous cell carcinoma: A preliminary study**. *Journal of the Chinese Medical Association : JCMA* 2022, **85**(1):95-101.

24. Zavridou M, Strati A, Bournakis E, Smilkou S, Tserpeli V, Lianidou E: **Prognostic Significance of Gene Expression and DNA Methylation Markers in Circulating Tumor Cells and Paired Plasma Derived Exosomes in Metastatic Castration Resistant Prostate Cancer**. *Cancers* 2021, **13**(4).

25. Basso U, Facchinetti A, Rossi E, Maruzzo M, Conteduca V, Aieta M, Massari F, Fraccon AP, Mucciarini C, Sava T *et al*: **Prognostic Role of Circulating Tumor Cells in Metastatic Renal Cell Carcinoma: A Large, Multicenter, Prospective Trial**. *The oncologist* 2021, **26**(9):740-750.

26. Ricordel C, Chaillot L, Vlachavas EI, Logotheti M, Jouannic A, Desvallees T, Lecuyer G, Aubry M, Kontogianni G, Mastrokalou C *et al*: **Genomic characteristics and clinical significance of CD56+ circulating tumor cells in small cell lung cancer**. *Scientific reports* 2023, **13**(1):3626.

27. Nastaly P, Ruf C, Becker P, Bednarz-Knoll N, Stoupiec M, Kavsur R, Isbarn H, Matthies C, Wagner W, Hoppner D *et al*: **Circulating tumor cells in patients with testicular germ cell tumors**. *Clinical cancer research : an official journal of the American Association for Cancer Research* 2014, **20**(14):3830-3841.

28. Qiu ZL, Wei WJ, Sun ZK, Shen CT, Song HJ, Zhang XY, Zhang GQ, Chen XY, Luo QY: **Circulating Tumor Cells Correlate with Clinicopathological Features and Outcomes in Differentiated Thyroid Cancer**. *Cellular physiology and biochemistry : international journal of experimental cellular physiology, biochemistry, and pharmacology* 2018, **48**(2):718-730.
